# Biomass-Derived Nanoporous Carbon Honeycomb Monoliths for Environmental Lipopolysaccharide Adsorption from Aqueous Media

**DOI:** 10.3390/ijms26030952

**Published:** 2025-01-23

**Authors:** Jakpar Jandosov, Dmitriy Berillo, Anil Misra, Mo Alavijeh, Dmitriy Chenchik, Alzhan Baimenov, Maria Bernardo, Seitkhan Azat, Zulkhair Mansurov, Joaquin Silvestre-Albero, Sergey Mikhalovsky

**Affiliations:** 1 Institute of Combustion Problems, 172 Bogenbay Batyr St., Almaty 050000, Kazakhstan; 2 Department of Chemistry and Chemical Technology, Al-Farabi Kazakh National University, 71 Al-Farabi Ave., Almaty 050012, Kazakhstan; 3 Department of Chemistry and Biochemical Engineering, Satbayev University, 22 Satbayev Ave., Almaty 050012, Kazakhstan; 4 Pharmidex Pharmaceutical Services, Fifth Floor, 167-169 Great Portland Street, London W1W 5PF, UKmo.alavijeh@pharmidex.com (M.A.); 5 School of Life and Medical Sciences, University of Hertfordshire, Hatfield AL10 9NE, UK; 6 Institute of Physics and Technology, 11 Ibragimov St., Almaty 050000, Kazakhstan; 7 Laboratory of Engineering Profile, Satbayev University, Almaty 050012, Kazakhstan; 8 LAQV/REQUIMTE, Departamento de Química (DQ), Faculdade de Ciências e Tecnologia (FCT), Universidade Nova de Lisboa (UNL), 2829-516 Caparica, Portugal; maria.b@fct.unl.pt; 9 Laboratorio de Materiales Avanzados, Departamento de Química Inorgánica, Universidad de Alicante, 03690 Alicante, Spain; 10 ANAMAD Ltd., Sussex Innovation Centre Science Park Square, Falmer, Brighton BN1 9SB, UK; sergeymikhalovsky@gmail.com; 11 Chuiko Institute of Surface Chemistry, National Academy of Sciences of Ukraine, 17 General Naumov Street, 03164 Kyiv, Ukraine

**Keywords:** carbon honeycomb monolith, rice husk, lignin, bacterial toxin, lipopolysaccharide, adsorption, water purification, point-of-use water treatment system

## Abstract

After undergoing biological treatment, wastewater still contains substances with endotoxic activity, such as lipopolysaccharide. However, due to the increasing practice of treating wastewater to make it suitable for drinking (potable reuse), the removal of these endotoxic active materials is crucial. These substances can be harmful to human health, leading to a condition called endotoxaemia. Furthermore, environmental endotoxins pose risks to pharmaceutical manufacturing processes and the quality of the final pharmaceutical products. Ultimately, the most significant concern lies with the patient, as exposure to such substances can have adverse effects on their health and well-being. Activated carbon has a proven efficiency for endotoxin removal; rice husk (RH), as a type of natural lignocellulosic agricultural waste, is a unique carbon precursor material in terms of its availability, large-scale world production (over 140 million tons annually), and is characterized by the presence of nanoscale silica phytoliths, which serve as a template to create additional meso/macropore space within the nanoscale range. High surface area RH/lignin-derived honeycomb monoliths were prepared in this study via extrusion, followed by carbonization and physical and chemical activation to develop additional pore space. The nanoporosity of the carbon honeycomb monoliths was established by means of low-temperature nitrogen adsorption studies, using calculations based on QSDFT equilibrium and BJH models, as well as mercury intrusion porosimetry (MIP) and SEM investigations. An alternative method for the elimination of the bacterial lipopolysaccharide (LPS)—a conventional marker—using filtration in flowing recirculation systems and the adsorbent activity of the monoliths towards LPS was investigated. Since LPS expresses strong toxic effects even at very low concentrations, e.g., below 10 EU/mL, its removal even in minute amounts is essential. It was found that monoliths are able to eliminate biologically relevant LPS levels, e.g., adsorption removal within 5, 30, 60, 90, and 120 min of circulation reached the values of 49.8, 74.1, 85.4, 91.3%, and 91.6%, respectively.

## 1. Introduction

Accessible clean water for everyone is one of the global challenges highlighted by the United Nations and included on the list of the seventeen UN Sustainable Development Goals for 2030 [1]. Continuous engineering development, agricultural expansion, and significant population growth place high demands on water supply, as well as creating new environmental and health threats associated with the pollution caused by the contaminants of emerging concern. Modern water management methods cannot always provide safe treatment to address these pollution challenges, and there is a necessity to improve current advanced technologies to meet existing demands [2].

Bacterial toxins are highly toxic substances produced by certain types of bacteria and they comprise two groups of toxins, according to their origin, e.g., endotoxins and exotoxins. In the literature, the terms “endotoxin” and bacterial lipopolysaccharide (LPS) are often used as synonyms, but not always. Occasionally, endotoxin refers to the Lipid A part of LPS or refers to an entirely different molecule that is released from cells only upon lysis. For this reason, LPS (or endotoxin) has been discussed to be classified rather as an (exogenous) hormone than as a toxin in a strict sense [3].

The World Health Organization (WHO) expressed its concerns about the lack of universal guidelines for endotoxin levels in drinking water. Water sources were reported to have different levels of endotoxins, with the range from 18 to 356 EU/mL in raw fresh water; 1 to 200 EU/mL in groundwater; 60 to 205 EU/mL in tap water; and from 4 to 119 EU/mL in drinking water [4].

LPSs endotoxins are ubiquitous in the living environment; they are the most common pyrogens (fever-inducing substances) found in pharmaceutical preparations. If entering the blood even in minute amounts, LPS’s endotoxins can cause tachycardia, fever, septic shock, tissue injury, multiple organ failure, and, ultimately, death. Since bacteria can grow in nutrient-poor media, such as water, saline, and buffers, LPS’s endotoxins are found almost everywhere. In conditions where the body is exposed to LPS’s endotoxins excessively or systematically (as when small concentrations of LPS continuously enter the blood stream), a systemic inflammatory reaction syndrome (SIRS) can occur, leading to multiple pathophysiological effects [5]. Generally, LPS endotoxins in water are found in two forms—free LPS endotoxins (i.e., dissolved in cell-free water) and bound endotoxins that are associated with viable bacterial cells and other suspended particles [6]. LPS’s endotoxins are relatively heat stable for one hour at 121 °C [7]. The process of LPS endotoxin removal from water during treatment processes at drinking water treatment plants (DWTPs) presents substantial challenges. Although the maximum LPS endotoxin limit in drinking water has not been established yet [8], there is a limit of no more than 0.25 IU/mL for the water for injections required by the European Pharmacopeia [9]. Even trace amounts of LPS endotoxins, at concentrations of less than 0.05 ng mL^−1^, in biopharmaceutical solutions and in the blood are able to activate monocytes and can induce a pyrogenic response in the human body by triggering the immune system’s signaling cascade, or the so-called cytokine storm, which is similar to that documented in patients with acute COVID-19 symptoms [10].

Endotoxic lipopolysaccharide (LPS) lipid complexes are found in the outer membrane (OM) of the cell walls of certain Gram-negative bacteria, such as anaerobic *Escherichia coli* (*E. coli*), *Shigella* and *Salmonella* spp., aerobic antibiotic-resistant *Pseudomonas aeruginosa* (*P. aeruginosa*), and cyanobacteria [11,12,13]. *E. coli* is classified as a rod-shaped, Gram-negative bacterium in the family *Enterobacteriaceae*. The bacterium mainly inhabits the lower intestinal tract of warm-blooded animals, including humans, and is often discharged into the environment through feces and wastewater effluent. The presence of *E. coli* in environmental waters within a wide pH range, e.g., 6–9, has long been considered as an indicator of recent fecal pollution. However, numerous recent studies have reported that some specific strains of *E. coli* can survive for long periods of time, and potentially reproduce, in extra-intestinal environments. This indicates that *E. coli* can be integrated into indigenous microbial communities in the environment [14]. *P. aeruginosa* is another ubiquitous bacterial species that colonizes various niches in the soil and the aquatic environment, especially in locations closely linked to human activity. Furthermore, it can survive for months in a wide range of temperatures and minimal nutrient requirements due to its good adaptation. The environmental sample screening identified *P. aeruginosa* as being significantly associated with hydrocarbon- and pesticide-contaminated environments, fecal sludge, and wastewater. Although various locations, such as water baths, showers, and swimming pools, are significant sources of *P. aeruginosa*, in most cases, antibiotic-resistant infections still occur in health care facilities, intensive care units, nursing homes, and hospitals, where immunocompromised and vulnerable individuals reside [12,15].

Some bacterial exotoxins, such as cyanotoxins, are secreted by cyanobacteria, or so called “blue-green algae”, which are microscopic organisms that are found naturally in all types of water, and which can sometimes cause cyanobacterial “algae” bloom. The cyanotoxins family includes not only neurotoxins, cytotoxins, and hepatotoxins, but also cyanobacterial LPS molecules that are also called “endotoxins” or “irritant toxins” because of their dermato-toxic and inflammatory properties, causing strong allergic reactions, as well as skin and eye irritations [13]. Some cyanotoxins have long been associated with adverse health effects, such as acute inflammatory gastrointestinal diseases, skin and eye irritation, vomiting, fever, and abdominal pain in humans, which are most likely obtained through drinking water or recreational activities [13,16,17]. It is no surprise, therefore, that “exogenous” bacterial LPS endotoxins derived from the destroyed cell membranes of both Gram-negative and cyanobacteria can often be found in the wastewater at DWTPs [18,19].

Several methods have been developed to eliminate or at least diminish the bacterial contamination of treated water; they include the infusion of chlorine after the activated carbon filter and prior to reverse osmosis. The contamination of dialysate by bacterial pyrogens is currently a serious problem in hemodialysis. Ideally, a dialysis fluid of pharmacological quality should be similar to that of infusion fluids. Therefore, in the manufacture of pharmaceuticals, such as water for injection (liquid pharmaceuticals that are directly introduced into the body) and medical devices (syringes, artificial organs, dialysis membranes, etc.), the strict control of endotoxins is critical. In this sense, efficient water treatment devices equipped with filters for the sterile filtration of dialysis fluid are indispensable [20,21].

The LPS endotoxin surface water levels range from 57 to 187 EU/mL depending on the location of the treatment plants [8]. Conventional oxidation processes for water disinfection using Cl_2_ or ClO_2_ were ineffective for endotoxin denaturation. Other conventional oxidation processes for water disinfection alone increased free endotoxin levels due to the lysis and oxidation of the bacterial cell surface [22]. Depyrogenation techniques, such as dry heating and chemical process, are not recommended, owing to changes in the chemical nature of the endotoxin molecules, as well as the formation of new by-products, which become a major challenge [4]. It was reported that coagulation/flocculation/sedimentation after chlorine pre-oxidation removed 76–85% of bound endotoxins. The drawback of such treatment is bacterial lysis and an increased level (by 28–33%) of free endotoxins detected in raw waters [8]. The depyrogenation of liquid preparations may be accomplished by filtration through various types of filter media including microporous membranes, reverse osmosis, membranes, ultrafilters, charge-modified depth filters, activated carbon (AC), and membrane absorbers [23]. The efficient removal of endotoxins from lysed *E. coli* media by depth filtration using a non-woven anion exchange material was reported [24].

Rice husk (RH) is an abundant agricultural by-product; its estimated annual world production is over 140 million tons [25]. In rice-growing countries, rice husk has minimal commercial value and it has traditionally been incinerated or disposed of to landfill. Rice husk is not readily decomposed by bacteria and its incineration causes air pollution, which makes the search for a more environmentally friendly means of its utilization vital [26]. In rice-producing countries, RH is used as a fuel. However, this product is characterized by low calorific value (13–15 MJ/kg) [27] and high ash content, most of which is composed of silica [28,29]. Converting RH into activated carbon, which has good adsorption properties, could not only add value to the product, yielding a useful carbon/silica composite material, but also reduce the inherent problems associated with the current methods of disposal of this waste by-product [25,29,30]. The nanoscale silica phytoliths contained in rice husk serve as a template to create additional meso/macropore space within the nanoscale range [28,31,32,33].

Recently, there has been a renewed interest in utilizing RH-based activated carbon as a low-cost sorbent for removing heavy metals and organic molecules from aqueous media [29,30,34]. In terms of potential advantages, it is a renewable bioavailable material with a low content of potentially toxic substances, which is important for biomedical applications.

Converting agricultural waste into activated carbon not only adds substantial value to the product with its potential use in adsorption and catalysis [35], but also reduces the inherent problems associated with the current methods of this biomass waste management, offering a “greener technology” approach.

Depth filtration, using activated carbon as a filter aid adsorbent, removes color, odor, and bacterial endotoxins. Its high adsorption capacity makes AC depth filtration an attractive alternative to other filtration methods. Powdered porous carbons (PCs) are widely used as adsorbents in purification and decolorization processes during the manufacture of pharmaceuticals. However, the use of powders can present an array of handling issues, where care must be taken to remove fine carbon particulates from the effluent. The use of mechanically robust carbon monoliths as filters has potential benefits, as neither the leaching of fine particulates or the need for the PC separation for disposal or regeneration is expected or required [36].

In this study, we explored the possible use of honeycomb carbon/silica monoliths obtained from RH powder as an adsorbent for the elimination of bacterial toxins from aqueous media using a bacterial lipopolysaccharide. Carbon monoliths with the honeycomb structure were produced in a cylindrical shape. They contained longitudinal channels, enabling a liquid flow with a minimal pressure drop and flow resistance, and meso/macropores located in the channel walls, which ensured their high adsorption capacity for LPS. LPS in the blood stream could trigger a huge inflammatory response, causing the release of a wide array of inflammatory cytokines and leading to the development of potential life-threatening conditions [37]. Previously, it was demonstrated that carbon sieves could adsorb LPS mainly on the external (macropore) surface [38]. Later, it was shown that carbon monoliths produced from phenol–formaldehyde resins with well-developed mesopores (2–50 nm) and macropores (>50 nm) effectively removed inflammatory cytokines and protein bound uremic toxins from human blood plasma in vitro [36]. The carbonized RH composites in the form of powder or monolithic column were also shown to adsorb a variety of medium and large-molecular-weight biological toxins such as LPS, and protein-bound uremic toxins, such as indoxyl sulfate and para-cresyl sulfate [39,40].

The goal of this work is to design a multifunctional activated carbon (AC)-based adsorbent and assess its efficiency in removing LPS—a model freshwater harmful algal bloom-derived cyanotoxin—from water, exploiting a novel carbon-honeycomb-structured technology, using sustainable green siliceous lignocellulosic components of a renewable resource (rice husk and lignin).

## 2. Results and Discussion

### 2.1. Synthesis and Structure of Rice Husk–Lignin-Derived Honeycomb Carbon Monoliths

According to Guo et al. [26], the constituents of RH are xylan (the main compound of hemicellulose) at 18.20 wt.%, cellulose at 35.86 wt.%, lignin at 24.52 wt.%, ash (mainly SiO_2_) at 18.85 wt.%, and other substances at 2.57 wt.%.

**Figure 1:**
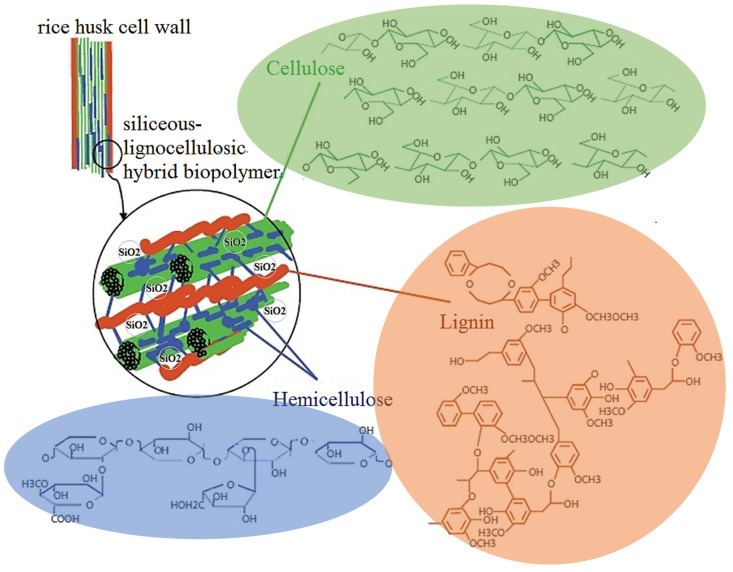
Structural components of rice husk cell wall, as a siliceous–lignocellulosic natural hybrid biopolymer.

Therefore, as a carbon composite precursor, RH could also be considered as a siliceous–lignocellulosic natural hybrid biopolymer nanocomposite. The structural components of the RH cell wall are shown in Figure 1.

Lignin is the second, after cellulose, most abundant natural raw material and nature’s most abundant aromatic (phenolic) biopolymer located in plant cell walls and the intercellular spaces of plants, whose main function is to cement the cellulose fibers in plants. Together with hemicelluloses, it determines the mechanical strength of trunks and stems [41,42].

Lignin, as a natural polymeric product, arises from an enzyme-initiated dehydrogenative polymerization of the three primary precursors or derivatives of hydroxycinnamic alcohols, i.e., p-coumaryl, coniferyl, and sinapyl alcohols, as shown in Figure 1. It is an integral part of lignocellulosic materials with the relative amount present varying over the approximate range of 10–30% [41].

**Figure 2:**
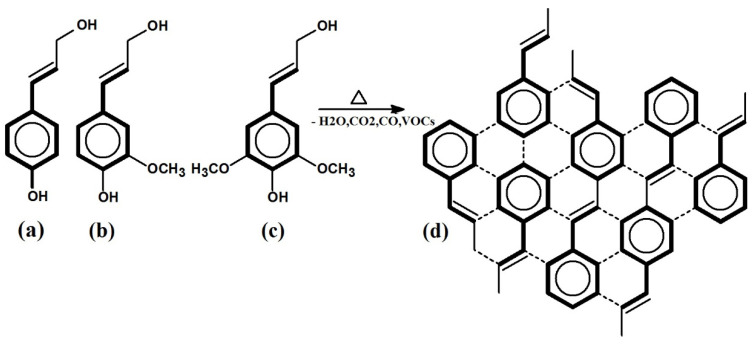
Formation scheme of graphitic layers from pyrolytic conversion via the polycondensation of lignin structural components, i.e., (**a**) p-coumaryl, (**b**) coniferyl, and (**c**) sinapyl alcohols; (**d**)—graphitic layer.(VOCs—volatile organic compounds, such as hydrocarbons, acetic and formic acids, aldehydes, and ketones).

A possible scheme for the graphite-like structure formation during the pyrolytic processes via the polycondensation of the structural components of lignin—three types of propylphenol, i.e., p coumaryl, coniferyl, and sinapyl alcohol—is shown in Figure 2.

Cellulose comprises 7000–15,000 glucose molecules and is a linear crystalline polymer. Hemicellulose comprises 500–3000 glucose units in an amorphous branched structure [42]. Both polysaccharides can be converted to carbon, although the weight yield is typically low—<15% in the absence of acid catalysts—and this gives rise to a low-density amorphous carbon.

Based on the analysis of the carbonaceous materials’ pyrolysis, three main processes and mechanisms can be distinguished, as follows: (1) the cracking and dehydrogenation of non-aromatic molecules; (2) the cyclization of hydrocarbon chains with n ≥ 6 into aromatics with the termination of side chains; and (3) the polycondensation of aromatic hexagonal structural fragments into more stable polycyclic arenes. Taken together, these reactions of scission and synthesis with recombination and consolidation lead to the formation of graphene-like layers. At the same time, these reactions are accompanied by the volatilization of part of the carbon in the form of gaseous by-products (VOCs). A scheme of possible chemical transformations during the carbonization of cellulose is shown in Figure S1 [43].

The production of carbons from any lingocellulosic precursor must take into account the fact that these materials comprise three separate components—cellulose, hemicellulose and lignin—which give rise to very different carbon materials.

The lignin component differs from cellulose and hemicellulose and is basically a phenolic polymer, as shown in Figure 2. This is produced from hydroxycinnamic alcohols, and the physical properties of the lignin depend on the proportions of these, which vary with the precursor. Lignins, like phenolic resin, give high carbon yields, typically around 50% weight, but half melting points and viscosities that vary with the source and the ratio of the phenolic components. In the production of activated carbon from lignocellulosic precursors, these two components—cellulose and lignin—therefore behave very differently. This has a major impact on the cellulose components, but has little or no impact on the phenolic (lignin) component. The process will therefore lead to a heterogenous material (C/C composite) with aspects of the structure deriving from both components.

In the case of carbon–silica composites, the use of alkaline agents such as NaOH or KOH is an effective way to develop mesoporosity in the matrix via alkali leaching and washing out the water-soluble Na or K silicates formed according to the reaction in Equation (1):

2MeOH + SiO_2_→Me_2_SiO_3_ + H_2_O (1)

where Me is Na or K. The natural inclusions of silica in the form of phytoliths in the native RH serve as a template for additional pore space formation [25,44].

Three types of carbon monoliths were synthesized from RH and lignin. They are denoted with letters C (carbonized), CA (CO_2_-activated), and CD (desilicated) according to the method of synthesis described in Section 3.

### 2.2. Physicochemical Investigation of Rice Husk-Derived Honeycomb Carbon Monoliths

#### 2.2.1. Low-Temperature Nitrogen Adsorption and Mercury Intrusion Porosimetry

**Figure 3:**
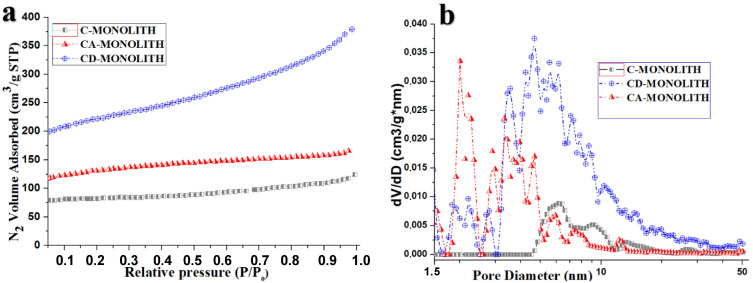
Surface area parameters of adsorbent RH–lignin-derived carbon monoliths. C (carbonized), CA (CO_2_-activated), and CD (desilicated). (**a**) N_2_ adsorption isotherms and (**b**) QSDFT pore size distribution.

The nitrogen adsorption isotherms are shown in Figure 3a. The pore size distribution of RH–lignin-derived carbon monoliths (Figure 3b) was calculated from nitrogen adsorption isotherms using the slit-cylindrical quenched solid density functional theory (QSDFT) equilibrium model. The S-shape of the nitrogen adsorption isotherm of the monolith-CD indicates that multi-layer adsorption takes place, which, according to the classification proposed by S. Brunauer, L. Deming, W. Deming, and E. Teller (BDDT), belongs to the type II characteristic of microporous materials [45].

The carbonized C-monolith has a broad multimodal mesopore size distribution in the range of 4.5–35 nm (Figure 3b); its subsequent CO_2_ activation leads to a slight decrease in the mesopore volume in this region and an appearance of a series of additional pronounced micro- and mesopore peaks within the range of ≤0.7 nm, ≥2–2.5 nm, and 3–5 nm in the CA-monolith. The peak of the mesopores’ distribution at 2.5–5 nm for the CA-monolith is shifted to a larger and wider peak within the range of 4–20 nm for the CD-monolith (Figure 3b). The desilication of the C-monolith leads to a drastic overall increase in both micropore and mesopore volumes manifested in the multimodal pore size distribution of the CD-monolith. The surface characteristics of the monolith samples obtained from low-temperature nitrogen adsorption studies are summarized in Table 1. The adsorbent CD-monolith possesses significantly higher surface area and pore volumes compared to both the C-monolith and CA-monolith.

The BJH cumulative pore size distributions for the monoliths are shown in Figure S2. The CD-monolith has a broader mesopore size distribution with the emergence of a pronounced peak within the range from 3 to 6 nm, compared to the CA-monolith. The pore volume of the CD-monolith at 4 nm is 0.4 cm^3^/g compared to 0.1 cm^3^/g in the CA-monolith. The CD-monolith also has a significant amount of large mesopores and small macropores with a diameter larger than 50 nm.

**Figure 4:**
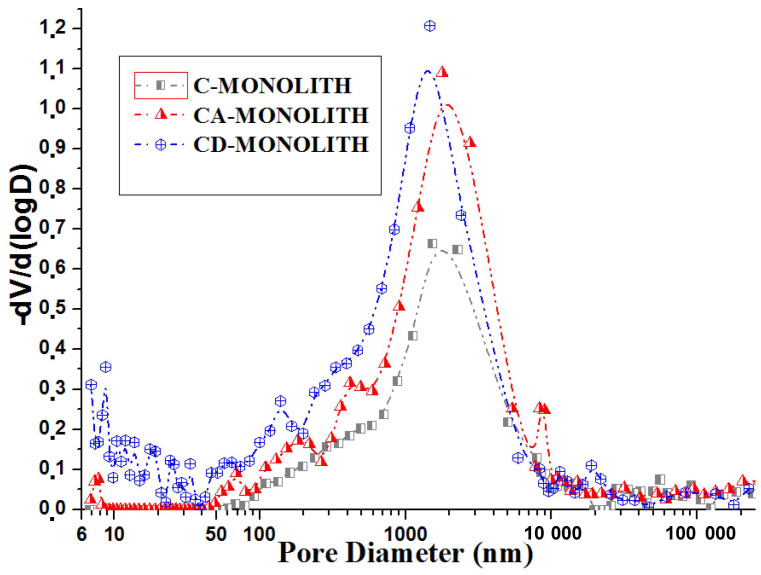
Pore size distribution in carbon monoliths according to the mercury intrusion porosimetry.

It can be concluded that the total pore volume increased dramatically in the CD-monolith versus CA-monoliths as a result of silica leaching during the alkali washing step (reaction Equation (1), Table 1, Figure 3 and Figure S2). Desilication due to the alkali leaching of the CA-monolith leads to a pore volume increase in the CD-monolith and a shift in the maximum mesopore volume from 3 nm to 4 nm. The mercury intrusion porosimetry pore size distribution of the monoliths is shown in Figure 4.

The graphs indicate the presence of pores with varying sizes in all the samples. A prominent peak in the range of 700–2000 nm signifies the existence of voids, likely formed due to the space between the fused carbon microparticles within the carbon monoliths and their longitudinal channels. Additionally, the desilicated CD-monolith exhibits a significant number of meso- and macropores in the range of 6 to 300 nm, categorized as meso-macropores. Compared to the C- and CA-monoliths (as shown in Table 1), the desilicated CD-monolith demonstrates a larger specific surface area and pore volume and was chosen for successive LPS adsorption studies.

#### 2.2.2. Investigation of the Internal Morphology of the CD-Monolith by SEM Imaging

**Figure 5:**
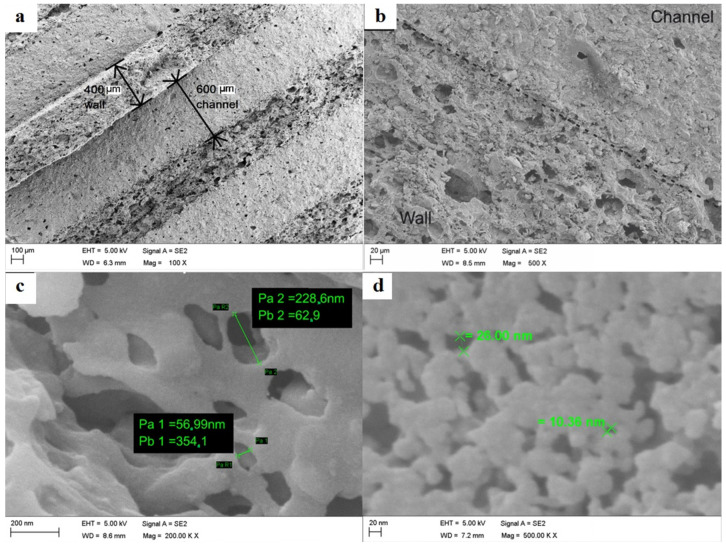
SEM micrographs of the CD-monolith obtained using a Zeiss Sigma field emission gun SEM. Magnification: ×100 (**a**), ×500 (**b**), ×200,000 (**c**), and ×500,000 (**d**).

The SEM images of the CD-monolith derived from rice husk and lignin binder displayed in Figure 5 reveal the porous nature of the material. The low-magnification images in Figure 5a,b show the main morphology of the monolith longitudinal section with extruded (wall) and internal (channel) structural differences visible, whereas the higher-magnification (Figure 5c,d) images demonstrate the main morphology of the porous internal structure in the small macropore and the mesopore diameter range, respectively.

The SEM images confirm that the surface morphology of the CD-monolith is characterized by a well-developed nano-(meso)porous and macroporous structure.

### 2.3. Bacterial Lipopolysaccharide Adsorption Studies

Owing to their highly developed internal surface area, which is suitable for the non-specific adsorption of predominantly hydrophobic, organic molecules, porous carbon (PC) adsorbents outperform other adsorbents in liquid phase applications. However, the use of granular PC formulations is constrained by a high pressure drop in the continuous column flow-through processes and the poor adsorption of larger biomolecules [36]. It has been shown that the adsorption capacity for large biomolecules depends on the availability of mesopores that are wide enough to accommodate such adsorbates [38,40,46].

A honeycomb porous carbon monolith with a well-developed micro- and mesoporous structure is an attractive alternative to the use of powdered or granulated PC materials. This structure can be maintained, and its flow resistance and pressure drop substantially, and are reduced by the production of activated carbon monoliths from powdered micro/mesoporous PC by extrusion. The monolith constructs of a cylindrical shape have numerous longitudinal transport micro-channels, which facilitate the liquid flow. The monolith production could be scaled up with the ultimate goal of providing a potential augmentation strategy for the filtration/adsorption of large biomolecules in an aqueous phase [36].

#### 2.3.1. LPS Structure, Function, and Mode of Biochemical Action

In recent decades, the interest in understanding the morphological features of LPSs and their impact on immunological activation, detection, removal, and neutralization has risen immensely, which can be demonstrated by the fact that the term “endotoxin” achieved more than 5000 new hits, and the term “lipopolysaccharide” achieved 7800 hits on PubMed in 2022. However, the broad heterogeneity of these molecules imposes a multi-layered challenge in comparing different studies [47]. LPS is also known as an endotoxin because the Lipid A that is released into the bloodstream of mammals can play a key role in pathogenesis and stimulates the innate immune system via triggering the Toll-like receptor 4/myeloid differentiation factor 2 (TLR4/MD-2) receptor signal transduction pathway, inducing a proinflammatory cytokine cascade and effects that may include hyperthermia, endotoxemia, and septic shock [47,48,49]. LPSs fulfill two major functions; first, the anchor in the OM provides a protective function for Gram-negative bacteria and, therefore, acts as a defense mechanism against harsh environmental conditions. Secondly, it provides a barrier against surrounding stress factors, which therefore makes LPS indispensable for bacterial viability in various distinct ecosystems [49].

**Figure 6:**
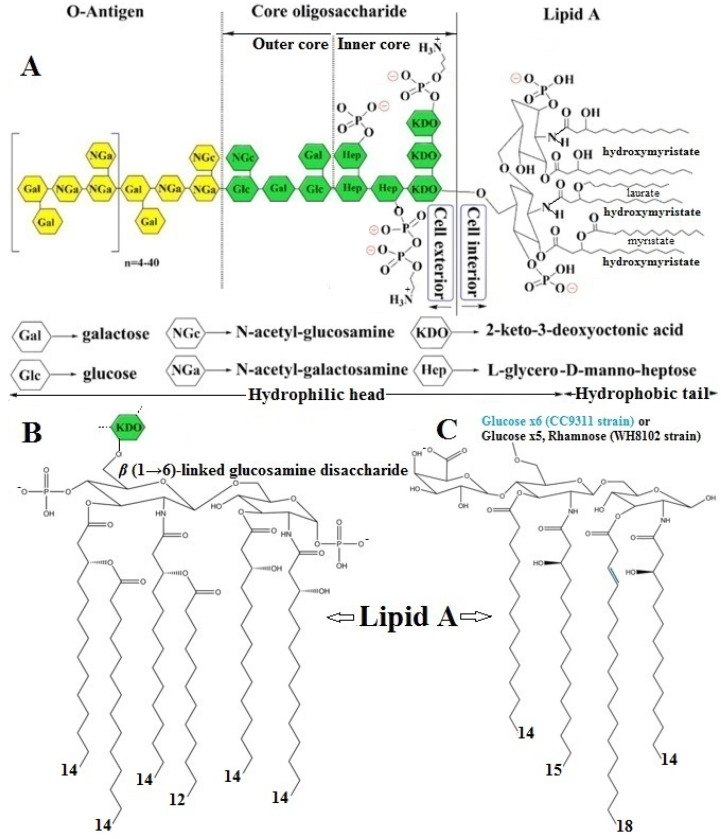
Schematic chemical structure of (**A**) *E. coli* LPS; (**B**) LPS Lipid A moiety of *E. coli*, and (**C**) *Synechococcus*, adapted from Tapouk et al. [4], Magalhães et al. [5], and Durai et al. [13].

Gram-negative bacterial LPS is an unacylated and phosphorylated macromolecular glycolipid that is anchored in the OM of the cell wall; it is composed of three genetically, biologically, and chemically distinct domainsthat are differentiated from one another, as shown in Figure 6A, based on their structure and function [5,13,47,48,49,50,51,52].

(1) A hydrophilic “core oligosaccharide” chain linkage region (core antigen or R antigen) is associated with immunogenicity and is generally composed of about 10 sugars and can be divided into an inner and outer region. The outer core region covalently binds an O-antigen moiety and contains common sugars, such as hexoses or partially N-acetylated hexosamines, whereas the inner core region, covalently bonded to the Lipid A moiety, is highly conserved across bacterial species and contains the partially phosphorylated unusual sugars, such as L-glycero-D-manno heptose and 3-deoxy-D-manno-octulosonic acid, also known as keto-deoxyoctulosonate (KDO) [47,52].
(2) A periodic hydrophilic antibody-binding O-antigenic (somatic) polysaccharide side chain that extends out into the environment is highly variable depending on the bacterial serotype, e.g., species and strain. The optional “O-antigen” consists of a variety of around eight different sugar moieties, usually hexoses, or sometimes partially N-acetylated hexosamines, including a repetitive polysaccharide monomeric subunit of 3–5 sugars that can build a chain of up to 50 repeats [47,52].
(3) A long, as well as bifurcated, hydrophilic carbohydrate and hydrophobic lipid moiety, termed“Lipid A”, is attached to the outer O-antigen carbohydrate chain via a“core oligosaccharide” linker and is mainly accountable for the proinflammatory endotoxic activity of the LPS molecule. The innermost Lipid A has enormous architectural diversity, but is extremely conserved across bacterial species and commonly has up to two (bi)-phosphorylated β(1→6)-linked glucosamine disaccharide backbones, which are mostly phosphorylated at position 1 and 4′ of the saccharides and acylated at positions 2 and 3 of each monosaccharide portion, connected with two to nine fatty acyl chains, typically composed of 10–16 carbon atoms each, through ester or amide linkages, anchoring the LPS molecule to the OM of the cell wall. The Lipid A glucosamine disaccharide of *E. coli* and *Salmonella typhimurium* spp. is linked to hydroxy fatty acids, such as hydroxymyristic acid, that are further acylated by nonhydroxylated fatty acids, such as myristic (tetradecanoic) and lauric (dodecanoic) acids [47,49,51] (Figure 6B).

The barrier function of LPS stems, in part, from its strong amphipathic nature. As in other lipid bilayers, the acyl portion of Lipid A provides a hydrophobic character that inhibits the passage of hydrophilic molecules through the OM. However, in contrast to other bilayers, the core oligosaccharide and O-antigen additionally provide extensive hydrophilic character to the LPS, which makes the OM particularly impermeable to hydrophobic compounds [52]. There are two main types of LPSs depending on the structural composition—the smooth type (S-LPS) and the rough type (R-LPS). The former is the complete form of an LPS with all three domains present, while the latter lacks the O-antigen domain. While Lipid A provides LPS with immunological and endotoxic properties, the role of the saccharide portion of LPS is also substantial [13]. The number of fatty acids is one of the major determinants of the immunogenicity of endotoxins. The most active form of Lipid A contains six fatty acyl groups and is found in pathogenic bacteria such as *E. Coli* and *Salmonella species* (Figures S3 and S4) [47,51]. Hexa-acylated Lipid A, such as *P. aeruginosa* (Figure S5) [51], seems to promote the strongest proinflammatory immune reactions after binding to TLR4 [49]. Not only do acyl chains contribute to the immunogenic properties of Lipid A, but also to the number of phosphate groups. The deletion of a phosphate group can result in a loss of the endotoxic activity. Most Lipid A structures contain two phosphate residues, one on each sugar moiety. However, some bacterial LPSs lack one or even both of them, therefore altering its immunogenic potential. For example, *Francisella tularensis* lacks one or both phosphate groups and this is considered to be, at least in part, the reason for its weak agonistic property (Figure S6) [47,49]. Underacylated Lipid A structures containing four or five fatty acids induce markedly less host defense responses and can inhibit the strong endotoxic response triggered by hexa-acylated LPS in a dose-dependent manner. However, certain bacterial species can provide “underacylated” Lipid A structures, like tetraacylated *Helicobacter pylori* Lipid A, or even “overacylated” structures like heptaacylated *Acinetobacter* spp. Lipid A (Figures S7 and S8) [47,49].

Most of the changes to the Lipid A structure occur in response to the amount of cations and positively charged antimicrobials in the environment, i.e., in the presence of cationic antimicrobial peptides (CAMPs) and host interactions. The most frequent modifications include changes in the number of phosphates in Lipid A, as well as the addition of covalent modifications to Lipid A, generally at the 1 and 4′ phosphates via phosphorylation, e.g., the covalent modification of Lipid A with 4-amino-4-deoxy-L-arabinose (L-Ara4N) and phosphoethanolamine (PEtN).Both the addition of positively charged moieties to Lipid A by L-Ara4N and PEtN, as well as the loss of the negatively charged modification, are associated with an increased resistance to CAMPs, presumably due to the masking of negative charges (i.e., phosphates) in LPS to which CAMPs bind [52]. Furthermore, the non-phosphorylation might also be seen as an immune evasion strategy for certain bacteria since some CAMPs specifically target negative charges on bacterial surfaces, and a loss of negatively charged phosphate residues therefore renders bacteria to be invisible for these CAMPs [49].

The LPS molecules from aquatic and terrestrial bacteria show structural variations, even among strains within the same species living in the same environment. Unlike Gram-negative bacterial LPS, cyanobacterial LPS has a unique structure, since it lacks heptose and KDO, which are present in the core region of common Gram-negative LPSs. In addition, the cyanobacterial Lipid A region usually lacks phosphates (except for *Microcistis* sp.) and contains odd-chain-hydroxylated or unsaturated fatty acid hydrophobic tails. A few structures of cyanobacterial LPSs have been reported; the quantity of key constituents in the LPSs of several cyanobacterial species is given in Table S1 [13]. The marine cyanobacterial LPS molecules of *Synechococcus* strains WH8102 and CC9311 have neither heptose nor KDO (Figure 6C), yet they have an α1,4-linked glucose chain; however, while WH8102 has a single rhamnose, the core region of CC9311 only consists of glucose [13]. Marine cyanobacterial LPS structures (or structural components) have been identified in *Synechococcus*, *Microcystis*, *Anacystis*, *Agmenellum*, *Shizothrix*, *Anabaena*, *Spirulina*, and *Oscillatoria* species. All of the species reported to date make unique LPSs, and presumably have unique LPS synthesis and modifying enzymes. Some of these structures include long fatty acyl chains with varying degrees of unsaturation; most lack phosphate modifications, such as in *Synechococcus*. Thus far, most of the structures reported have not been directly supported in the literature with genomic, transcriptomic, or proteomic data [48]. In their review, Jakubowska et al. pointed out that cyanobacteria have a modified form of LPS that is structurally distinct from common LPS and is known to be less toxic [13]; however, the toxicity of cyanobacterial LPS is only five times lower than that of the LPS of *E. coli*. The LD_50_ of the cyanobacterial LPS (in mice) ranges from 40 to 190 mg kg^−1^ and depends on the cyanobacteria species. Like the terrestrial Gram-negative LPS, cyanobacterial LPS can be toxic to humans by causing allergies or respiratory and skin diseases [53].

In the late 1970s, Keleti et al. reported that the most common cyanobacterium contaminating drinking water systems during the epidemic algal bloom in southwestern Pennsylvania (1979) was *Schizothrix calcicole*, which has less than 0.1% of phosphorus in its LPS content; this epidemic was concomitant to the maximal concentrations of algae and cyanobacteria observed in the raw water. The team concluded that all types of bacterial LPS may cause endotoxemia, especially in debilitated, immunosuppressed patients and infants, e.g., enhanced gastrointestinal permeability in infants suggests that the absorption of exogenously derived endotoxins (from milk, water, etc.) could initiate illnesses under certain conditions. At that time, the presence of endotoxins in drinking water and the effect of LPS’s being introduced into the bloodstream have been observed indirectly; the pyrogenic reactions among kidney dialysis patients were established at that same time [54]. In the early 1980s, Keleti et al. isolated LPS from the cyanobacteria *Anabaena cylindrica* and *Oscillatoria brevis*, which frequently occur in drinking water supplies. Unlike Lipid A from heterotrophic Gram-negative bacteria, lipidA from cyanobacteria usually lacks phosphates. Endotoxins from *Anabaena cylindrica* and *Oscillatoria brevis* were toxic to mice when injected intraperitoneally. The cyanobacterial endotoxins showed a generally lower biological activity than that of LPS derived from common heterotrophic Gram-negative bacteria. And yet, experiments with adrenalectomized mice and bioassays with *Oscillatoria brevis* and *Anabaena cylindrica* LPSs suggested that the toxicity of cyanobacterial endotoxins may only be approximately at levels 10 times lower than that of heterotrophic Gram-negative bacteria, such as Gram-negative *Salmonella* sp.(Figure S4) [51]. Nevertheless, cyanobacteria in algal blooms may be a significant source of endotoxins in water supplies [55]. Since bacteria are omnipresent in the environment, an immense and constant load of LPS molecules is released into our surroundings, which becomes especially dangerous during an injury or a bacterial infection, when these organisms transpose our epithelial barrier [47]. Candelli et al. suggested that LPSs can trigger chronic inflammatory bowel diseases (IBDs), where the source of LPS is contained in water or food and is usually sustained by the gut microbiota (GM); metabolic endotoxemia is typically present in IBD, which boosts gut enterocytes’ permeability, causing the so-called “leaky gut” syndrome [56]. A study by Šindlerová et al. established that the accidental consumption of contaminated water during recreational activities in freshwater cyanobacterial harmful bloom (Cyano HAB)-affected reservoirs may expose, in a mixture of other bioactive molecules and/or cyanotoxins, enterocytes to LPSs from Cyano HABs. The LPSs can contribute to the activation of the enterocytes and to disruptions of the intestinal epithelium. An impaired intestinal epithelium can result in the increased paracellular transport of LPSs, exposing immune cells residing in the near proximity. LPSs were isolated from water blooms dominated by *Microcystis aeruginosa* (*M. aeruginosa*), *Aphanizomenon klebahnii*, *P. agardhii*, *Oscillatoria platoprix*, and *D. curvum*, which are common bloom-forming cyanobacterial species in Czech Republic water bodies. The authors concluded that complex environmental mixtures of cyanobacterial water bloom LPSs can have proinflammatory effects on the intestinal epithelium, as well as on immune cells in vitro [57]. In this context, chronic exposure to LPSs, which leads to IBD development, has also been linked to the development of disorders such as Alzheimer’s disease [56]. Hence, the toxicological investigations of Mayer et al. concluded that the cyanobacterial LPSs of *Scytonema javanicum* and *Scytonema ocellatum*, as well as of *M. aeruginosa* and *Oscillatoria* spp., elicit the release of reactive oxygen species, such as the superoxide anion, generated by rat microglia in vitro and that can cause neuronal injury via oxidative stress, which have been implicated in neurodegenerative diseases; the study contributes to understanding of the underestimated potential toxicity of cyanobacterial LPSs on the brain immune system of mammals, including humans [58].

#### 2.3.2. Justification of LPS Concentration

Endotoxin levels reported in raw water samples worldwide varied in a wide range of concentrations, from the lowest of 9 EU∙mL^−1^ to the highest of 356 EU×mL^−1^ [6,8,18,59]. Rapalaet al. measured endotoxin concentrations at nine different full-scale drinking water treatments plants in Finland that were experiencing toxic blue–green algae (cyanobacteria) blooms in 2002. They found that endotoxin concentrations ranged from 18 to 356 EU/mL at the plant intakes, and 3 to 15 EU/mL in the finished water in the distribution system [18,59].

Can et al. recorded relatively low endotoxin concentrations (41 EU×mL^−1^) in the source water of a DWTP in Beijing, compared to its concentration in the source water of DWTPs in Wuhan (86–101 EU×mL^−1^) [6]. Endotoxin concentrations measured in 69 point-of-use water treatment systems and tap waters in South Korea varied within the ranges of 0.8–79.1 EU×mL^−1^ and 0.1–3.4 EU×mL^−1^, respectively [60]. Can et al. found that the endotoxin level dropped from 21–31 EU×mL^−1^ in source water to 4–10 EU×mL^−1^ after DWTP treatment, showing a 31% decrease in free endotoxins and a 71% decrease in bound endotoxins throughout the entire water purification process [6]. Interestingly, endotoxin activity increased after a combined treatment with granulated activated carbon and chlorination, probably due to the induced endotoxin release following bacterial cell disruption. A similar observation of an increased endotoxin activity following water treatment with biological AC filtration, ozonation, and chlorination was reported [59]. The authors suggested that in addition to cell disruption, a possible cause of this activity elevation was the release of Gram-negative bacteria inhabiting the carbon filter. However, rapid sand filtration after these processes effectively reduced the endotoxin level.

In this context, for our studies, we have chosen an initial endotoxin concentration of about 25 EU×mL^−1^, which exceeds the upper end of its common concentration levels in tap and drinking water in developed countries.

#### 2.3.3. Adsorption of LPSs Using RH–Lignin-Derived Carbonized–Desilicated Honeycomb Monoliths

Since LPS Lipid A possesses two distinct structural functional groups, e.g., hydrophobic lipo- and phosphate-anionic regional groups, two different approaches can be considered for its elimination. This can make it challenging to compare and assess the degree of elimination and its mechanism. For instance, the elimination process could involve the adsorption of the hydrophobic hydrocarbon molecular region of Lipid A. Alternatively, it might involve anionic phosphate ion exchange with the assistance of functionalized polymeric resins, graphene oxide derivatives, amino multi-walled carbon nanotubes, or similar adsorbents. However, it is essential to be mindful of the potential drawbacks, such as low mechanical strength and nanotoxicity. In this context, two approaches are possible—one involving low-cost, high-quality carbon materials for removing LPSs from aqueous environments and the other employing highly sophisticated and expensive methods like ion exchange or ultrafiltration based on size exclusion, among others.

**Figure 7:**
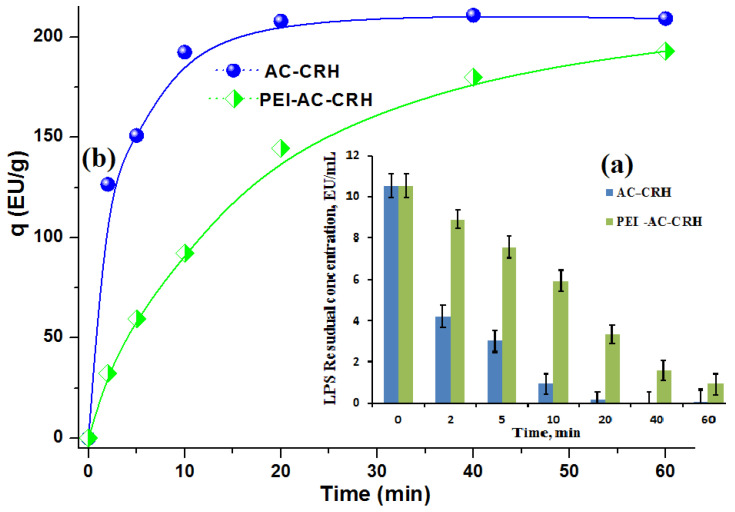
LPS adsorption profiles for (PEI-) AC-CRH carbon adsorbents. (**a**) Remaining concentration decrease over adsorption time course; (**b**) adsorption capacity (q) increase during 1 h of incubation in PBS.

Previously, we conducted LPS batch adsorption studies using carbonized and steam-activated RH (AC-CRH adsorbent) [39]. AC-CRH adsorbent subsequently underwent cationization of the surface with polyethyleneimine (PEI) to yield PEI-AC-CRH adsorbent, which, in fact, led to a slight decrease in adsorption capacity (q) and adsorption kinetics, e.g.: from 209.3 EU×g^−1^ for AC-CRH to 193.12 EU×g^−1^ for PEI-AC-CRH adsorbent, probably due to AC-CRH surface inhibition by PEI. LPS adsorption profiles for AC-CRH and PEI-modified AC-CRH adsorbents are sown in Figure 7a for the remaining concentration decrease over adsorption time course and in Figure 7b for the adsorption capacity increase during 1 h of incubation in phosphate-buffered saline (PBS); a comparison is also given in Table 2.

Analyzing the data given in Table 1, Figure 3, Figure 4 and Figure S2, it can be concluded that the total pore volume increased dramatically in the CD-monolith versus the CA-monoliths as a result of silica leaching during the alkali washing step (Equation (1)), compared to the C- and CA-monoliths (as shown in Table 1), the desilicated CD-monolith demonstrates larger specific surface area and micro/meso/macropore volumes.

**Figure 8:**
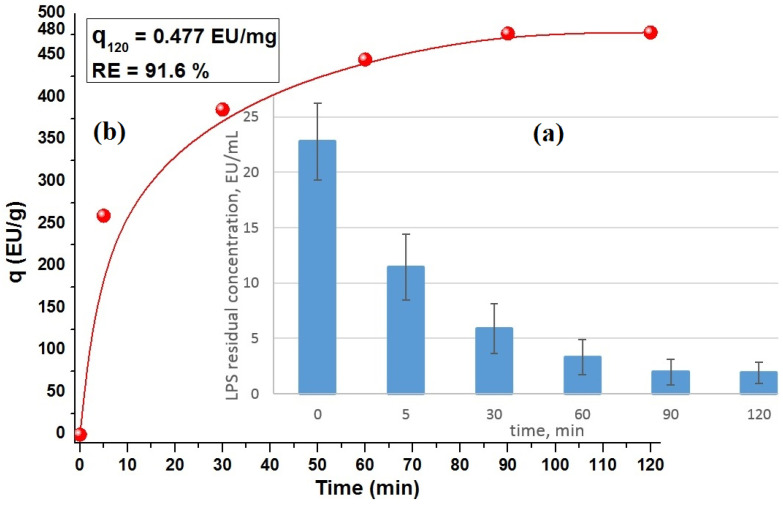
LPS adsorption profiles for carbon honeycomb CD-monolith. (**a**) Remaining concentration decrease over adsorption time course; (**b**) adsorption capacity (q) increase during 2 h of recirculation.

The adsorption profiles of LPS from PBS with the CD-monolith are shown in Figure 8, demonstrating the high efficiency of LPS removal.

The current research adsorption profiles of LPS in PBS with the CD-monolith shown in Figure 8 demonstrate a high efficiency of LPS removal.

There are various adsorbents that have been studied for their ability to remove LPSs, including carbon nanomaterials, silica, chitosan, and amino-compounds, e.g., dimethylamine, amino acids, Polymixin B, etc. Although a direct comparison may be subjective due to different experimental conditions, it allows for a perception about the performance of the developed adsorbents. A comparison between the performance of different adsorbents used for LPS removal concerning their adsorption capacity is shown in Table 2.

Recently, Tapouk et al. prepared a graphene oxide (GO)-based adsorbent using epichlorohydrin (ECH) as a coupling agent and dimethylamine (DMA) as a ligand for LPS removal from aqueous solutions [4]. The obtained GO-ECH-DMA adsorbent significantly removed 98% of LPSs in solution with an adsorption capacity (q_e_) of 12,147 EU/g. Additionally, the GO-ECH-DMA adsorbent could be regenerated five times during theadsorption–desorption cycles with no significant loss in its adsorption capacity. Furthermore, the adsorption mechanism revealed that the excellent adsorption efficacy of the GO-HCH-DMA adsorbent might be attributed to a combination of hydrogen bonding, π-π stacking, and electrostatic interaction. Despite the advantages, e.g., high efficiency, unique complex structure of LPS, and diverse adsorption mechanisms, the obvious disadvantages are the costly chemicals needed, the tedious process, a preliminary batch adsorption study, the possible low mechanical strength in ongoing column studies, and nanotoxicity due to the leakage of fine particulates [4].

Similarly, Zong et al. prepared novel polyvinyl alcohol-amino multi-walled carbon nanotube (PVA-AMWCNT) nanocomposite microspheres and PVA-polymyxin B (PMB)—a cationic polypeptide antibiotic—nanocomposite microspheres [61]. The authors pointed out that PMB can cause nephrotoxicity and neurotoxicity if it leaks off the matrix. AMWCNT and PMB amino groups were coupled with the preliminary activated PVA microsphere matrix, using epichlorohydrin cross-linker in alkaline dimethyl sulfoxide solution, followed by sonication in polyvinyl pyrrolidone solution. The results of comparative LPS batch adsorption studies revealed that the LPS adsorption capacity of PVA-AMWCNT composite microspheres increased almost tenfold compared to that of unmodified PVA microspheres, and slightly increased over that of PVA-PMB composite microspheres, e.g., 114 EU/g vs. 108 EU/g [61].

Hou et al. [62] and Wei et al. [63] designed a series of affinity chromatography columns packed with agarose beads, as a non-specific matrix, functionalized with different amino acids ligands; hexamethylendiamine was used as a spacer; and epichlorohydrin was used as a cross-linker. In batch adsorption studies of LPS removal from aqueous solutions, these selective adsorbents exhibited a high adsorption capacity of 152 [62] and 292.4 [63] EU/g, respectively. The columns were designed for LPS elimination via hemoperfusion; the preliminary studies showed good removal rates and biocompatibility. On the other hand, these types of adsorbents are barely suitable for water purification from LPS on a large scale, except for pre-dialysis chromatography, because of the use of high-cost materials, the sophisticated manufacturing process, and the susceptibility of agarose to microbial attack.

Cao et al. fabricated cross-linked cellulose microspheres (CL-CMs) for applications in blood purification using PMB as a selective cationic ligand for LPS removal [64]. Epichlorohydrin was used to enhance the mechanical strength of the gel microspheres by cross-linking. Cellulose gel microspheres possess high surface areas, a good spherical shape, and monodispersity. The adsorbent showed excellent LPS removal from physiologic saline solution with the maximum adsorption capacity of 3605 EU/g; the removal rate exceed 70% [64]. Despite the fact that cellulose—an abundant, low-cost, natural, biodegradable, and non-toxic polysaccharide—was used as a matrix, the drawback is that both the physical and chemical immobilization of PMB was involved during the preparation process of CL-CMs-PMB adsorbent, and if desorbed, PMB can cause serious side effects to the human body.

Huang et al. designed 3D electro-spun hybrid nanofiber sponges prepared from polyacrylonitrile (PAN) and SiO_2_ using freeze-drying in order to obtain a good stability of PAN and a good biocompatibility of SiO_2_, which was derived from tetraethylorthosilicate [65]. The matrix exhibited a high specific surface area and porosity, and PMB was successfully immobilized onto its surface at 110mg/g using 1-(3-Dimethylaminopropyl)-3-ethylcarbodiimide as a cross-linker. The authors theorized that the protonated diaminobutyric acid residues of PMB may be ionically bound to the monophosphate or diphosphate group of LPS Lipid A. The adsorption performance of the PAN-SiO_2_-PMB nanofiber sponge adsorbent was investigated in different solutions. The adsorption capacity in physiological saline solution was around 17.9 EU/mL, and the adsorption equilibrium time was about 40 min. The endotoxin removal rate in human plasma reached 90%, and the adsorption reached equilibrium within 60 min, which proved the PMB immobilized-nanofiber sponge would have great potential for applications in clinical blood purification [65]. The advantages are its high removal efficiency and mechanical stability, while the disadvantages are its technical complexity and high reagent costs.

Li et al. successfully fabricated homogeneous chitosan-kappa-carrageenan composite hydrogels for LPS removal from the blood via phase inversion and genipin cross-linking techniques. The cross-linked hydrogels demonstrated satisfactory mechanical strength and a porous microstructure, with the outer carrageenan shells contributing to an enhanced antifouling property. The resulting adsorbent displayed a remarkable LPS adsorption capacity, with values of 114 EU/g in simulative septic blood and 203 EU/g in PBS. The endotoxin removal efficiency reached 63% during a 3h simulative hemoperfusion procedure [66]. Despite the widespread use of chitosan and kappa-carrageenan in the food and pharmaceutical industries due to their natural origin, abundant amino groups, and cost-effectiveness, it is important to note that the cross-linking agent used—genipin—is an exotic natural terpenoid and is considerably expensive. Therefore, it seems to be difficult to scale up such a composite adsorbent for water purification.

Du et al. prepared activated charcoal cross-linked agarose-coated carbon adsorbents (CAAC-II) for endotoxin removal from model isotonic saline via perfusion through a small column, which held 2 g of CAAC-II. The volume of endotoxin solution (1000 pg mL^−1^ at pH 7.0) was 20 mL; the recirculating time was 120 min. The maximum removal efficiency achieved was 69%; the adsorption capacity at 120 min was 58 EU/g [67]. The advantages are inexpensive reagents and simple experimental setup; the disadvantages are the relatively small values of adsorption capacity, the removal efficiency, and the susceptibility of agarose to bacterial attack.

Rezaee et al. prepared bone char adsorbent via cattle animal bone pyrolysis in a furnace at 850 °C. The aim of this study was the removal of endotoxins from aqueous solution using bone char (BC) as an adsorbent material [68]. The maximum removal efficiency achieved was 98% after 6 h of adsorption time, while the equilibrium adsorption capacity was approximately 29 EU·g^−1^. The advantages are that the recovery of renewable and inexpensive adsorbent material is an important aspect of water and wastewater treatment. The regeneration of used BC has been carried out using a thermal process that was regenerated and rendered endotoxin-free by heating at 350 °C for 30 min [68]. The disadvantages are the relatively small values of adsorption capacity, as well as the BET surface area (130.7 m^2^/g), which is probably due to its high mineral content (hydroxyapatite).

The question about how LPS removal can be carried out in an economical way has attracted the attention of many investigators and has been the reason for process rearrangements in many cases. However, this issue has not yet been resolved satisfactorily. In the selective removal of LPS from protein-free solutions, it is easy to remove LPS by ultrafiltration by taking advantage of the different sizes of the LPS and water, or by an anion-exchanger or non-selective adsorption with a hydrophobic adsorbent [5].

#### 2.3.4. Relationship of LPS Adsorption with Textural Characteristics of Carbon Monoliths and Its Potential Use/Modification

Each bacterial species has its own pool of LPS molecules varying in their chemical composition and enabling aggregation into different supramolecular structures upon release from the bacterial cell wall. In general, amphiphilic molecules can be encountered in their monomeric form in diluted solutions; however, when the critical micellar concentration (CMC) is reached, the molecules tend to aggregate and form micelles. S-LPS strains tend to form aggregates at higher LPS concentrations than R-LPS strains. Additionally, it is expected that simple surfactants aggregate cooperatively and exhibit a narrow CMC, whereas the determination of the CMC for amphiphilic molecules with a broader molecular weight distribution is rather complex. CMCs ranged from 10 to 14 µg/mL with the presence of pre-micelles or evolving micelles until higher concentrations were reported [47]. The typical molecular weight of the monomeric S-form LPS of *E. coli* is reported to be between 11.8 and 18 kDa [49].

Brandenburg et al. have reported that the biologically most active part of enterobacterial LPS—hexaacyl bisphosphorylated Lipid A—adopts particular supramolecular conformations [69]. However, little is known about the size and morphology of these aggregates in relation to the fact that LPSs may have strong variations in the length of the saccharide chains (various R-LPS mutants and S-LPS forms). Their data show a variety of different morphologies not only for different endotoxins, but also when comparing different applied physicochemical techniques; electron microscopy studies revealed that deep R-LPS mutants from the resulting aggregates at room temperature are vesicle-like liposomes with diameters from about 50 to 200 nm, in addition to the spherical liposome-like structures with diameters in the range of 200–500 nm; analytical ultracentrifugation demonstrated the transformation for both samples, exhibiting a size distribution for S-LPS between 5 and 105 nm with peaks of the diameters at 120–180 nm, whereas the R-LPS mutant shows a much broader size distribution between 100 and 420 nm and between 100 and 700 nm. They concluded that the experimental data provide evidence for a very complex aggregation behavior of bacterial lipopolysaccharides, with structure formations deviating from spheres especially in the case of longer saccharide side chains. In particular, except for one publication, the detailed properties of aggregates, as well as their sizes and type of structures, in dependence on concentration down to the picomolar range, which is relevant in biological systems, are rarely characterized [69].

Gautier et al. [70] demonstrated that, as compared to S-LPS, R-LPS formed larger aggregates, with a higher hydrophobicity index, a more negative zeta potential, and a higher critical aggregation concentration. Their data indicate that a long polysaccharide chain is associated with the formation of more stable aggregates with an extended residence time in plasma and a higher inflammatory potential. S-LPS and R-LPS were dissolved in saline at identical concentrations (20 μmol/L) and were first analyzed by atomic force microscopy. Both LPS solutions contained aggregates. They were apparently homogeneous in each preparation but differed markedly from one LPS type to the other. S-LPS formed small, simple globular aggregates with an apparent diameter ranging from 20 to 30 nm. R-LPS formed larger aggregates (apparent diameter of 100–200 nm) with simple-to-multi-lobular spherical shapes. They concluded that polysaccharide chain length, zeta potential, and the overall aggregability of LPS should be considered when predicting the proinflammatory effect that can be expected in experimental settings [70].

**Figure 9:**
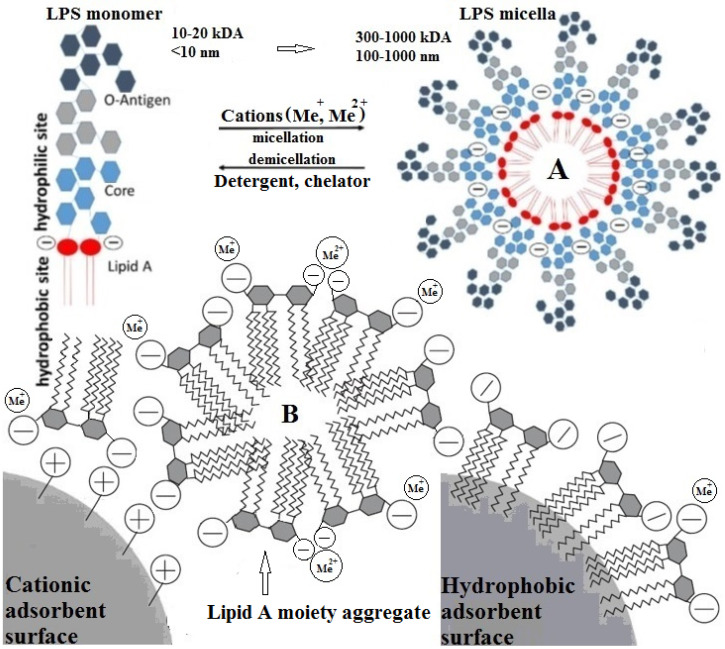
The proposed schematic mechanisms of amphiphilic LPS micellation–demicellation (**A**) and adsorption on cationic and hydrophobic biomaterial surfaces in aqueous solution media (**B**). Adapted from Harm et al. [71] and Gorbet and Sefton et al. [72]. Red colorrepresents hydrophobic part (lipid) of molecule, blue coloris ionichydrophilic part and grey colorrepresents carbohydrate moiety.

Harm et al. proposed that in aqueous solution, monomeric LPS molecules form aggregates as a supramolecular assembly because of its amphiphilic nature in the form of micelles or vesicles; Lipid A was inserted into the aggregate interior, with the hydrophilic sites as head groups [71]. Bivalent cations form bridges between the negatively charged phosphate groups in the lipid A fraction. By adding chelators (EDTA, citrate) and detergents (DOC, Tween), the LPS aggregates can be destabilized and disintegrate into monomers (Figure 9A). In aqueous solutions, endotoxins aggregate as a supramolecular assembly because of their amphiphilic nature.

Gorbet and Sefton et al. suggested that the phosphate groups of monomeric endotoxins can form the outer layer of this supramolecular assembly, and that the other parts form the inner one, which allows the endotoxin aggregates to interact with cationic adsorbents. Due to their hydrophobicity, endotoxins adsorb readily to hydrophobic materials and will also bind cationic materials through their phosphate groups [72] (see Figure 9B).

Wereszczynski et al. point out that there is no consensus on the charge state of phosphate groups within LPSs, and different force fields assign different net charges; *E. coli* LPSs determined the pKa values in solution as 8.6 for the first lipid A deprotonation and 10.8 for the second deprotonation, indicating that the fully protonated state charge of minus one per phosphate should dominate in solution at physiological pH. Based on these results, they proposed that the current LPS force field parameterizations should be updated to correct these charge inaccuracies and offer the following phosphate charge guidelines. At or near physiological pH, each LPS lipid A phosphate group should carry a charge of minus one [73].

There are many advantages in adapting the activated carbon form to a monolithic structure for direct flow-through filtration/adsorption, but also many challenges in maintaining a porous profile and adsorptive efficacy. Previous tests using a small monolith prototype and hemodialysis patient blood samples indicated that the adsorption of protein-bound and middle molecules was dependent on creating nanopores in the 2–100 nm range [36]. The challenge in scaling up the monolith is to maintain nanoporosity, but also to design the monolith channel parameters to balance maximum adsorption with appropriate internal fluid dynamics. This is achieved in the current device iteration by using a constant 0.6 mm microchannel channel size. A short diffusion distance is provided for access to the internal porous surface area in the walls of the monolith and a micro-channel structure capable of minimizing shear stress and maintaining laminar flow. The introduction of a monolithic design was therefore integral to minimize the tendency for flow irregularities and pressure drop [36].

From mercury intrusion porosimetry and low temperature adsorption studies (Table 1 and Figure 3 and Figure 4), it was shown that the CD-monolith has a great potential in having both large macro- and micro-mesopore total pore volumes for future adsorption studies at high LPS concentrations.

When it comes to the future insights regarding the LPS adsorption capacity enhancement using RH-derived carbon monoliths, an unsophisticated soft ammoxidation (oxidative ammonolysis) technique can be employed by means of ozonation, followed by ammonia treatment [40]. That will render both nonspecific hydrophobic adsorption via interaction of carbon surface with nonpolar fatty acyl chains of Lipid A, as well as the formation of amino groups on the carbon monoliths surface. These surface functional groups may act as CAMP-like species allowing the gain in overall LPS adsorption capacity via ionic interaction of positively charged (protonated) amino groups with the negatively charged phosphate anionic groups of Lipid A.

## 3. Conclusions

The study of morphological features of LPSs and their impact on immunological activation, detection, removal, and neutralization has garnered significant interest in recent decades. The method of the carbon monolith preparation with an additive of lignin was ascribed. However, the inherent heterogeneity of these molecules presents a complex challenge when comparing different research studies. To overcome this hurdle, the integration of various binding molecules capable of distinguishing LPS subtypes in a single assay holds the potential to revolutionize diagnostic tools and broaden their applications in the medical field. Notably, the carbon monoliths demonstrated remarkable LPS adsorption removal rates during a 2 h perfusion, with an initial LPS concentration typical of elevated levels observed in sepsis patients. After a 2 h recirculation, the LPS concentration was effectively reduced tenfold. In vitro adsorption studies of the RH–lignin-derived honeycomb monoliths in a flowing recirculation system provided encouraging results, indicating their ability to effectively remove significant levels of LPS from aqueous solutions. The adsorption removal efficiency rates reached the values of 49.8%, 74.1%, 85.4%, 91.3%, and 91.6% within 5, 30, 60, 90, and 120 min of circulation, respectively. The nanoporosity of the carbon honeycomb monoliths was comprehensively investigated, using low-temperature nitrogen adsorption studies, QSDFT equilibrium and BJH model calculations, mercury intrusion porosimetry, and SEM analysis. These analyses confirmed the presence of nanoporosity in the monoliths. Overall, the findings from this study shed light on the promising potential of nanoporous-rich carbon honeycomb monoliths in the removal of LPS from aqueous solutions, offering valuable insights for future medical applications, particularly in the management of sepsis and related conditions. Further research and exploration in this area hold great promise for advancing diagnostic and therapeutic strategies in the medical field.

## 4. Experimental

### 4.1. Materials and Methods

PEI (polyethyleneimine with an average MW of 2 kDa) was purchased from Sigma-Aldrich Co., Taufkirchen, Germany; NaOH 99%, PBS, and LPS endotoxin marker (*E. coli* 0111:B4, #L2630) were purchased from Sigma-Aldrich Ltd., (St. Louis, MO, USA); Pierce LAL assay (Limulus Amoebocyte Lysate Chrmogenic Endotoxin Quantitation Kit) was purchased from Thermo Fisher Scientific Inc. (Life Technologies Limited 3 Fountain Drive, Inchinnan Business Park, Paisley, UK). RH was collected from the rice fields of the Almaty region, Kazakhstan; Organosolv™ lignin was provided by MAST CARBON Ltd., Basingstoke, UK; METHOCEL™ K15M (a high-molecular-weight hydroxypropyl methylcellulose thickener) was purchased form (Colorcon Asia Pvt. Ltd., Verna, India); PEO-H (polyethylene oxide grade with a viscosity average molar mass (Mw) of 2 × 10^6^ g/mol) was purchased from VWR Prolabo (VWR, Rosny-sous-Bois, France).

### 4.2. Preparation of AC-CRH and PEI-AC-CRH Adsorbents

RH pyrolysis was carried out at 700 °C at a ramp rate of 2.5 °C/min with additional steam activation for 90 min. For such a carbonization technique, 500 cm^3^ of RH was loaded into a rotary steel reactor placed in an electrically heated furnace and equipped with a capillary injection system. The liquid water volume rate was maintained at 100 cm^3^/h to obtain AC-CRH adsorbent [74]. PEI-AC-CRH adsorbent was produced from AC-CRH adsorbent by incubation in 5.0 mM aqueous solution of PEI.

### 4.3. Preparation of Honeycomb Carbon Monoliths

Honeycomb carbon monoliths were obtained from milled RH powder (fraction: 40–63 µm) by mixing it with an organic carbonizable Organosolv™ lignin-based binder at the (*wt*/*wt*) ratio of RH/lignin3:1, which was then found to give the best balance between the strength and pore structure retention of the finished carbon monoliths. The initial “dough” mixture composition was as follows: milled RH (fraction 40–63 microns)—60 g; Organosolv™ lignin—20g; Methocel K15M—13.71 g; PEO-H—2.4 g; and distilled water—100 g.

The “dough” mixture thus prepared was extruded through a die plate (600 CPI and 10 mm diameter) using an Instron^®^ vertical small-scale extruder and the resulting “green” monoliths were cured in the fridge for 2 days. The matured monoliths were first stabilized by heating in air (letter box oven) from RT to 250 °C, before being heated up to 700 °C at a temperature ramp rate of 1 °C/min in a horizontal tube furnace under the steady flow of CO_2_ (1 L/min) and were carbonized for 20 min to produce C-monoliths (the weight loss is 65%).

A batch of carbonized C-monoliths was additionally activated under similar conditions in CO_2_ at 850 °C for 1.5 h to yield CA-monoliths (burn-off is 19%). Alternatively, a batch of C-monoliths underwent SiO_2_ extraction; the monoliths were immersed in 1M NaOH solution in a vertical position and were then heated up slowly to 80 °C and incubated for 2 h. The monoliths were base-leached twice to remove as much silica as possible and were subsequently washed with deionized water under reflux in a Soxhlet extractor until neutral pH and dried in ambient conditions to produce carbonized and desilicated CD-monoliths (the weight loss is 35%). See the monolith preparation technique on the scheme shown in Figure S8 [47] for the subsequent flow experiments; CD-monoliths were shrink wrapped by heat in Polyolefin Heat Shrink Cable Sleeve (RS Components Ltd., Corby, UK).

### 4.4. Honeycomb Carbon Monolith Geometry Design, Parameters, and Function

**Figure 10:**
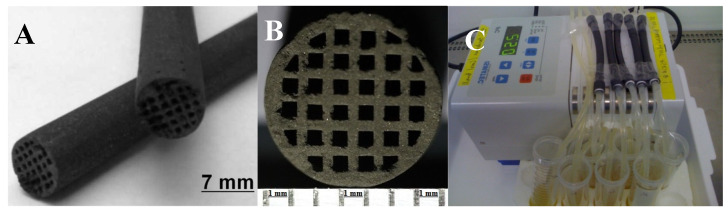
Honeycomb carbon CD-monoliths. (**A**) External appearance of as-prepared monolith-CD—10 cm length, 6.9 ± 0.1 mm diameter; (**B**) geometry of the cross-section area: number of channels—32; cell side length—0.6 mm; wall diameter—0.4 mm; (**C**) a flowing recirculation system for LPS adsorption studies of CD-monoliths.

The geometry and design of extruded “green” monoliths is defined by the type of dye plate; in our experiments, the initial “green” monoliths had a diameter of 10 mm, but their linear parameters decreased by around 30% due to shrinkage in the course of carbonization. The small, as-prepared, and cylindrical prototype CD-monoliths with the channel structure through which an aqueous LPS solution recirculates using a peristaltic pump are shown in Figure 10A–C.

The transverse section of the monolith-CD depicted in Figure 10B consists of 32 cells (24 square- and 8 triangular-shaped) within the circular area of 6.9 ± 0.1 mm in diameter, with a cell side length of 0.6 mm and a wall diameter of 0.4 mm. The shrink-wrapped 10-cm-long monolith was connected with standard sterile syringe tips to a peristaltic pump, and a PBS solution spiked with LPS was recirculated through the channels (Figure 10C). Assuming that the area of a triangular-shaped cell for 8 peripheral channels is approximately half as large as the area of a square cell, the total volume of channels (V_channels_) within a monolith was estimated according to Equation (2):

V_channels_ ≈ (m + 0.5n) × L × d^2^ (2)

where **m** is the number of square cells (24); **n**—the number of triangular-shaped cells (8); **L**—the length of a monolith (10 cm); and **d**—the cell diameter (0.6 mm; 0.06 cm). According to Equation (1), the volume of channels is approximately V_channels_ = 1.008 cm^3^ ≈ 1 mL.

The cylindrical volume of monolith-CD was assessed according to Equation (3):

V_monolith_= 0.25π d^2^ × L ≈ 3.85 mL (3)

The moisture capacity of the monolith-CD was calculated according to Equation (4):

W_monolith_= V_H2O_/(M_monolith_+ M_H2O_) ≈ 0.55 mL/g (4)

where W_monolith_ is the moisture capacity of the monolith-CD, M_H2O_ = V_H2O_ = 1.6 g (mL) is the mass/volume absorbed by the monolith-CD, and M_monolith_ = 1.31 g is the CD-monolith average mass.

### 4.5. Physical and Physicochemical Methods of Investigation

#### 4.5.1. Low-Temperature Nitrogen Adsorption Studies

The textural properties of the monolith samples were studied by the method of low-temperature nitrogen adsorption using a surface area and porosimetry analyzer “TriStar3000” (Micromeritics Inc., Norcross, GA, USA). Upon the preliminary outgassing of the samples at 150 °C for 3 h with a residual pressure of less than 0.001 mmHg, the nitrogen adsorption isotherms were recorded at the temperature of liquid nitrogen (77 °K) within the range of relative pressures from 0.005 to 0.995. The specific surface area was calculated using the Brunauer–Emmett–Teller (BET) theory. The Barret–Joyner–Halenda (BJH) method was used to calculate pore size distribution within the range from 1.7 to 300 nm from the adsorption branches of the isotherms. The quenched solid density functional theory (QSDFT), assuming a slit/cylindrical equilibrium pore model, was employed for both QSDFT total pore volume (VDFT) and surface area (SDFT) calculations, as well as QSDFT pore size distribution analysis.

#### 4.5.2. Mercury Intrusion Porosimetry

For mercury intrusion porosimetry analysis, the carbon adsorbent samples were degassed overnight in a vacuum oven at 150 °C. Mercury intrusion/extrusion curves, “isotherms”, were recorded using the Poremaster instrument (Quantachrome, Hook, UK). The data were analyzed using “Quantachrome Poremaster” data analysis PoreMaster Analysis Software (V8.01) to calculate the mesopore volume (<50 nm), as well as the meso/macropore volume (<100 nm) of the materials.

#### 4.5.3. Scanning Electron Microscopy Imaging

For SEM imaging, a monolith was cut into longitudinal sections to be able to view the internal carbon structure. The samples were mounted onto a sample holder and coated with a 4-nm-thick layer of platinum using a Quorum (Q150TES) coater. The sections were examined using a Zeiss Sigma field emission gun SEM (Zeiss, Jena, Germany) at an accelerating voltage of 5 kV.

### 4.6. In Vitro Adsorption of LPS-Endotoxins

#### 4.6.1. Batch Adsorption of LPS by AC-CRH and PEI-AC-CRH Samples

Time-course batch experiments were performed to establish the dynamics of LPS adsorption on (PEI-) AC-CRH adsorbents. In a typical set of experiments, 100 mL of 10 EU×mL^−1^ LPS solution in phosphate-buffered saline (PBS) with a pH of 7.4 was added to 5 g of an adsorbent in a 250 mL conical flask and incubated for an indicated time at 25 °C. The remaining LPS concentration was determined by taking aliquots (1 mL) at various times (2 min, 5 min, 10 min, 20 min, 40 min, and 60 min) using 5 mL pipettes. Each sample was centrifuged at 1000 rpm for 1 min to remove dust. Then, the concentration of LPS was measured using the pierce LAL assay.

#### 4.6.2. In Vitro Adsorption of LPS by Carbon CD-Monoliths

The studies on the adsorption of LPS markers by the carbon CD-monoliths were carried out in a flowing recirculation system equipped with a peristaltic pump set at a flow rate of 4 mL/min (Figure 10C). Endotoxin-free distilled water was used to make PBS solution and for sample dilution. The monoliths were primed with PBS solution for 2 h; in total, 25 mg of LPS was transferred into a depyrogenated glass vial with 10 mL of distilled water and reconstituted by vigorous shaking in a vortex mixer, before being diluted 1000-fold with distilled water; the resultant 45 μL aliquots were pipetted into Thermo Scientific™ Nunc™ 50 mL conical sterile polypropylene centrifuge tubes with45 mL of PBS solution each to give the initial LPS concentration of ca. 23 ± 7 EU×mL^−1^. The LPS-spiked PBS solution was continuously recirculated through the monoliths using a peristaltic pump model [36]. At the time points of 5, 30, 60, 90, and 120 min. of perfusion, 100 μL aliquots of the solution were collected in triplicate for the assessment of the remaining LPS concentration using the pierce LAL assay. The samples were diluted 1:500 with endotoxin-free distilled water, and their optical absorbance was measured at 405 nm using Tecan Safire 2 Multi-Mode Microplate Reader (Infinite^®^ F50 Magellan™ Software Magellan Version 6.3 (6.3.0.8) Tecan Austria GmbH Untersbergstr. 1A A-5082 Grödig, Austria).

The amount of endotoxin adsorbed per mass unit or adsorption capacity, *q_t_* (EU×mg^−1^), and endotoxin removal efficiency, *RE* (%), were computed using Equations (5) and (6), respectively [4]:

(5) $$q_{t}(\mathrm{EU}\times {\mathrm{mg}}^{-1})=\frac{\left(C_{0}-C_{t}\right)V}{w\;}$$

(6) $$RE\left(\%\right)=\frac{\left(C_{0}-C_{t}\right)}{C_{0}\;}\times 100$$

where *q_t_* (EU×mg^−1^) is the endotoxin amount adsorbed by the time *t*(min), *RE* (%) is the endotoxin removal efficiency (percentage), and *C*_0_ and *C_t_* are initial and current endotoxin concentration at the time *t* (EU×mL^−1^), respectively. *W* (mg) is the weight of adsorbent (mg), and *V* (mL) is the volume (mL) of the endotoxin solution.

## Figures and Tables

**Table 1:** Specific surface area (S) and pore volume (V) of carbon monoliths calculated using different methods *.

Sample Code	S_BET_,m^2^/g	S_DFT_,m^2^/g	V_DFT_,m^2^/g	D_DFT_, nm	V_DR_, cm^3^/g	V_BJH_, cm^3^/g	V_MIP≤100 nm_, cm^3^/g	V_MIP≤300nm_, cm^3^/g
C-Monolith	327	390	0.168	6.16	0.131	0.083	0.005	0.062
CA-Monolith	492	472	0.233	1.19	0.206	0.064	0.022	0.090
CD-Monolith	837	802	0.540	1.10	0.348	0.294	0.129	0.256

* BET—Brunauer–Emmett–Teller (BET) theory; DFT—quenched solid density functional theory; BJH—Barrett–Joiner–Halenda method; DR—Dubinin–Radushkevich equation; MIP—mercury intrusion porosimetry analysis.

**Table 2:** Comparison of various adsorbents for LPS adsorption.

Adsorbent Matrix	q* (EU×g^−1^)	q* (μg×g^−1^)	*RE (%)	Ref.
Dimethylamine-functionalized graphene oxide (GO-ECH-DMA)	12,147	1.215 × 10^1^	>98	[4]
PVA/amino multi-walled carbon nanotubes nanocomposite microsphere (PVA-AMWCNT)	114	1.14 × 10^−2^	>90	[61]
Aminoalkylagarose-hexamethylenediamine-L-lysine	152.2	1.522 × 10^−2^	73	[62]
Aminoalkylagarose-L-phenylalanine (His)	292.4	2.924 × 10^−2^	37.1	[63]
Cross-linked cellulose microspheres (CL-CMs)	3605	3.605 × 10^−1^	>70	[64]
Polymyxin B immobilized polyacrylonitrile/SiO_2_ nanofiber sponge	17.9	1.789× 10^1^	90	[65]
Carrageenan-immobilized genipin-cross-linked chitosan hydrogel	202.8	2.03 × 10^−2^	63	[66]
Cross-linked agarose-coated activated charcoal (CAAC-II)	58.2	5.82 × 10^−3^	69	[67]
Bone Char (BC)	28.8	2.88 × 10^−3^	98	[68]
AC CRH	209.3	2.09 × 10^−2^	>99	[39]
PEI-AC-CRH	193.1	1.931 × 10^−2^	91.5	Present work
Monolith-CD	477.3	4.77 × 10^−2^	91.6	Present work

q*—adsorption capacity; *RE—removal efficiency or sorption degree.

## Data Availability

All data are contained within the manuscript and are available upon request.

## Supplementary Materials

The following supporting information can be downloaded at: https://www.mdpi.com/article/10.3390/ijms26030952/s1. References [13,43,47,51] are cited in the Supplementary Materials.
