# Supplementary material for: Biomass-Derived Nanoporous Carbon Honeycomb Monoliths for Environmental Lipopolysaccharide Adsorption from Aqueous Media
Source: Int J Mol Sci. 2025 Jan 23;26(3):952. doi: 10.3390/ijms26030952 (PMC11817206; doi:10.3390/ijms26030952)
Supplement: Supplementary file 1 [file ijms-26-00952-s001.zip › ijms-3369302-supplementary.pdf]

Supplement information

Biomass-derived Nanoporous Carbon Honeycomb Monoliths for Environmental Lipopolysaccharide Adsorption from Aqueous Media

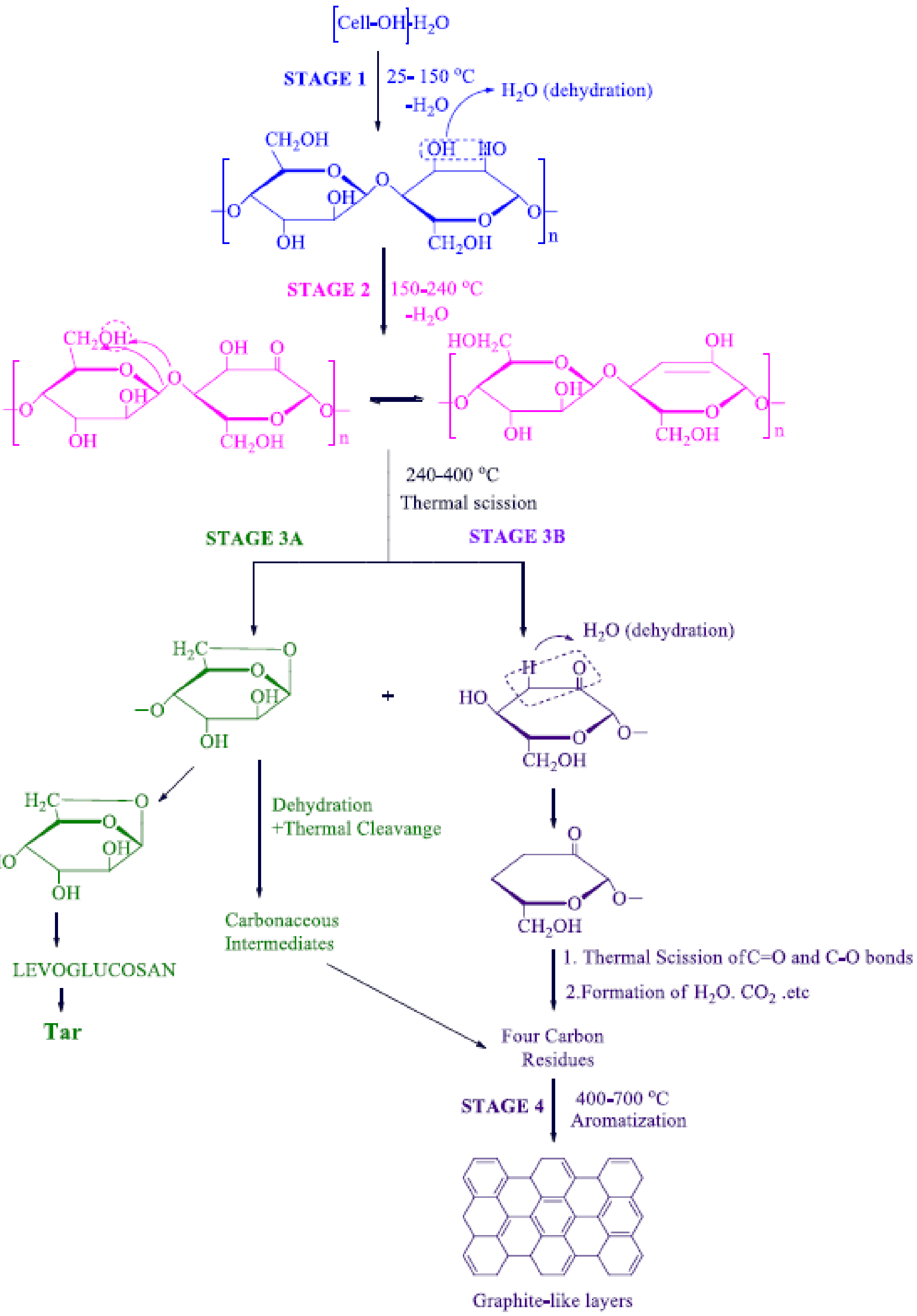

Figure S1. Scheme of possible chemical transformations during carbonization of cellulose [43]

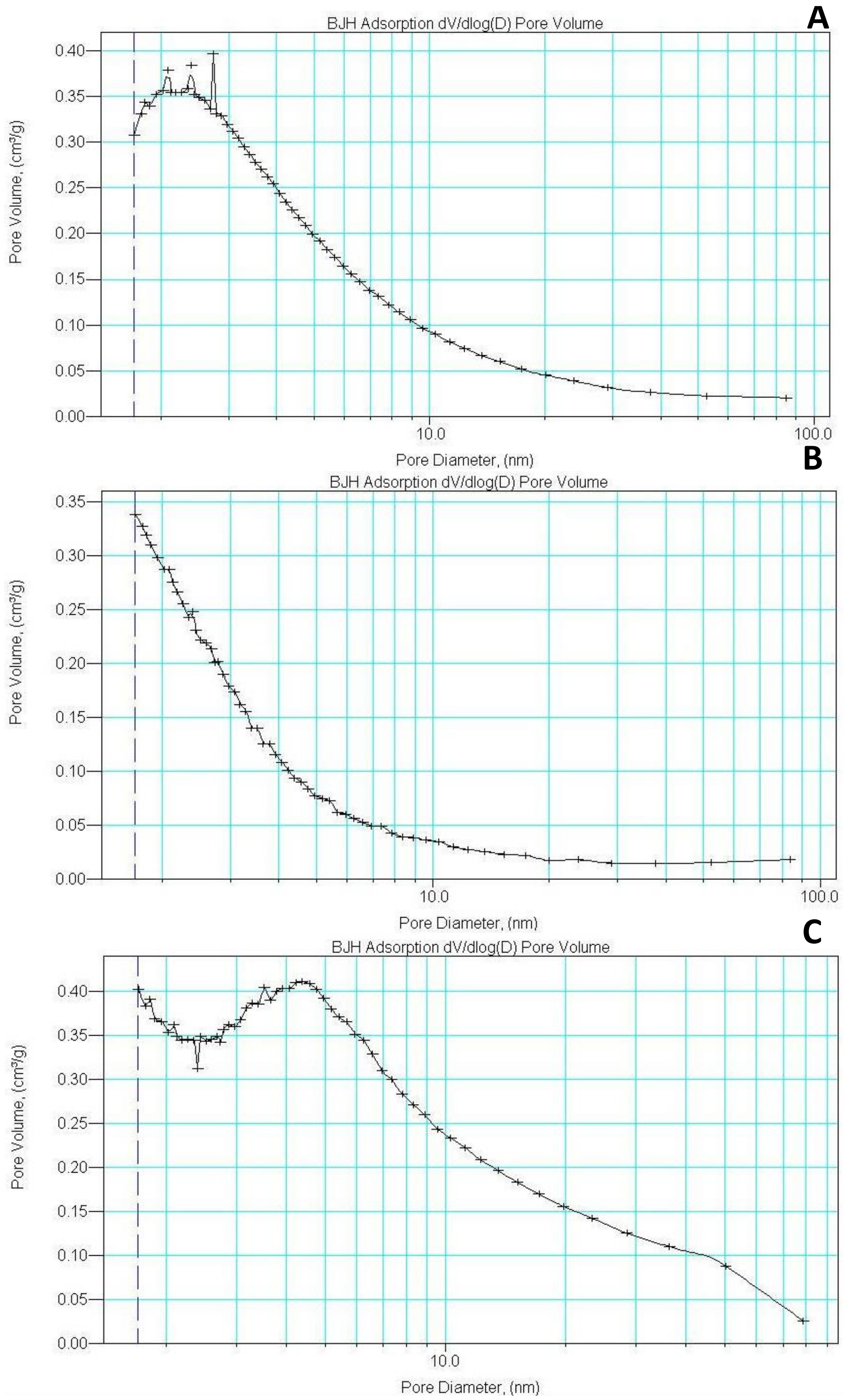

Figure S2. Cumulative pore size distribution of (a) C-(carbonized) carbon monolith; (b) CA-(CO_2-activated) carbon monolith and (c) CD- (desilicated) carbon monolith calculated using BJH method.

Table S1. The chemical composition of major components in cyanobacterial LPS [13]

Cyanobacterial Species	Carbohydrates (%)	Phosphorus (%)	KDO (%)	Proteins (%)	Fatty Acids (%)
Schizothrix calcicola	63	<0.1	Absent	7.8	8
Phormidium spp.	60	<1	0.5	7.20	NA
Agmenellum quadruplicatum	59.5	2.9	0.13	0.13	15.1
Anabaena variabilis	80.3	0.03	Absent	8.4	10.7
Spirulina platensis	31.6	0.6	NA	0.6	14.3
Anacystis nidulans	60	0.03	1.5	NA	12.4
Microcystis aeruginosa	36.0	0.7	Absent	0.4	18.2
Anabaena flos-aquae	65	Absent	12.5	NA	NA

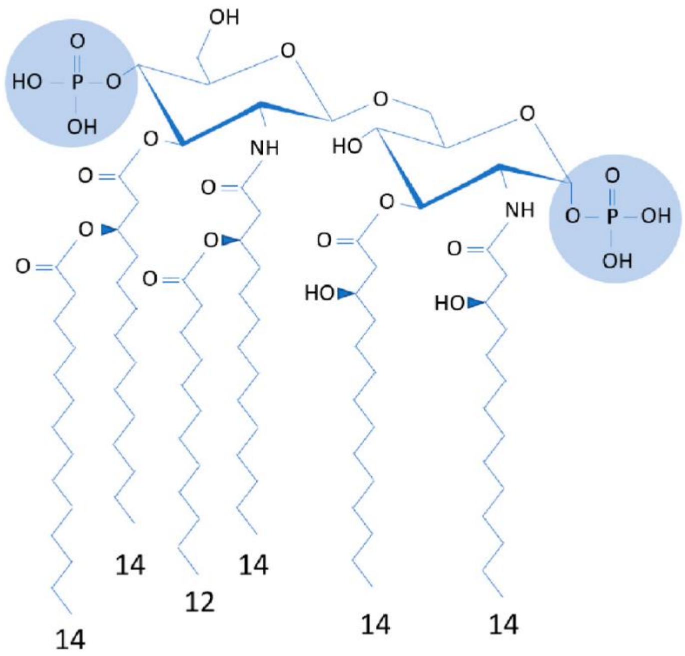

Figure S3. Chemical structures of LPS produced by:*Escherichia coli* [47]

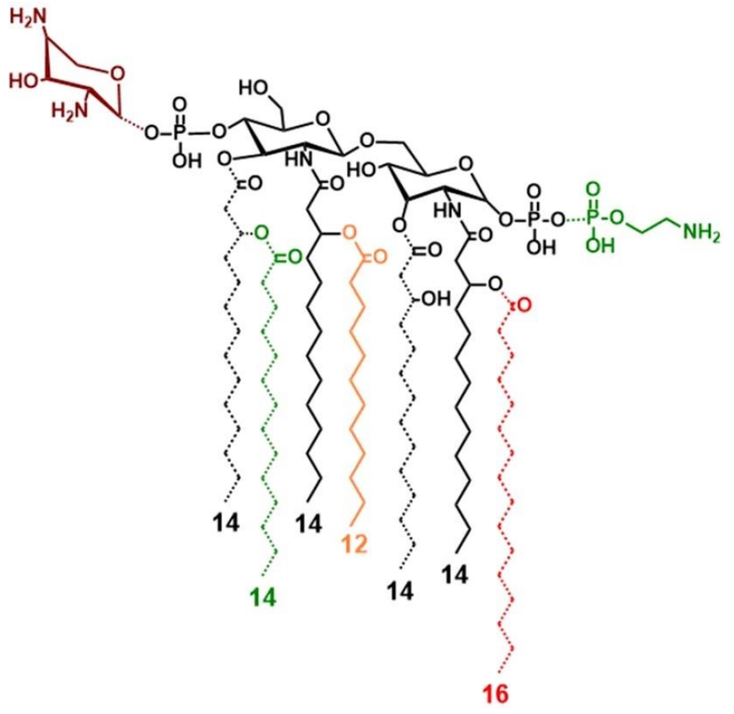

Figure S4. Chemical structures of LPS produced by : *Salmonella typhimurium* [51]

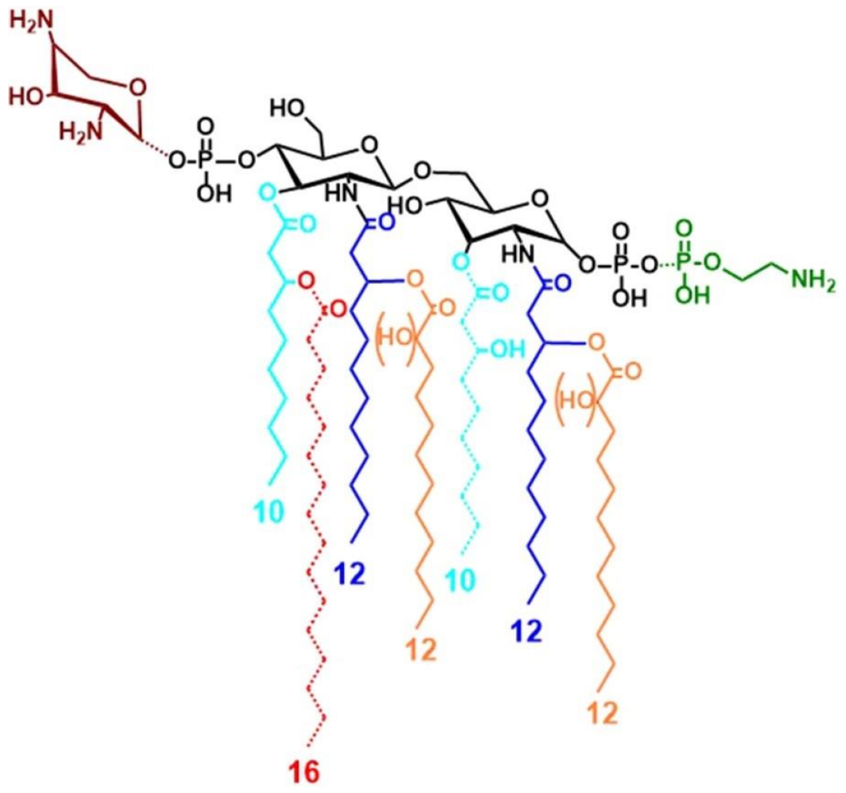

Figure S5. Chemical structures of LPS produced by *Pseudomonas aeruginosa* [51]

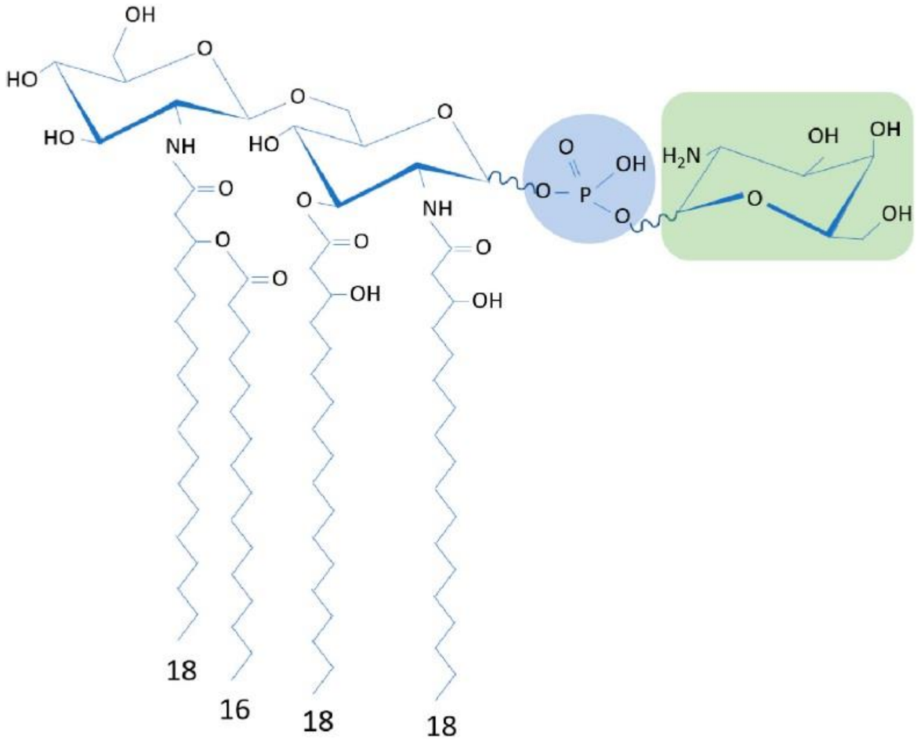

Figure S6. Chemical structures of LPS produced by *Francisella tularensis* [47]

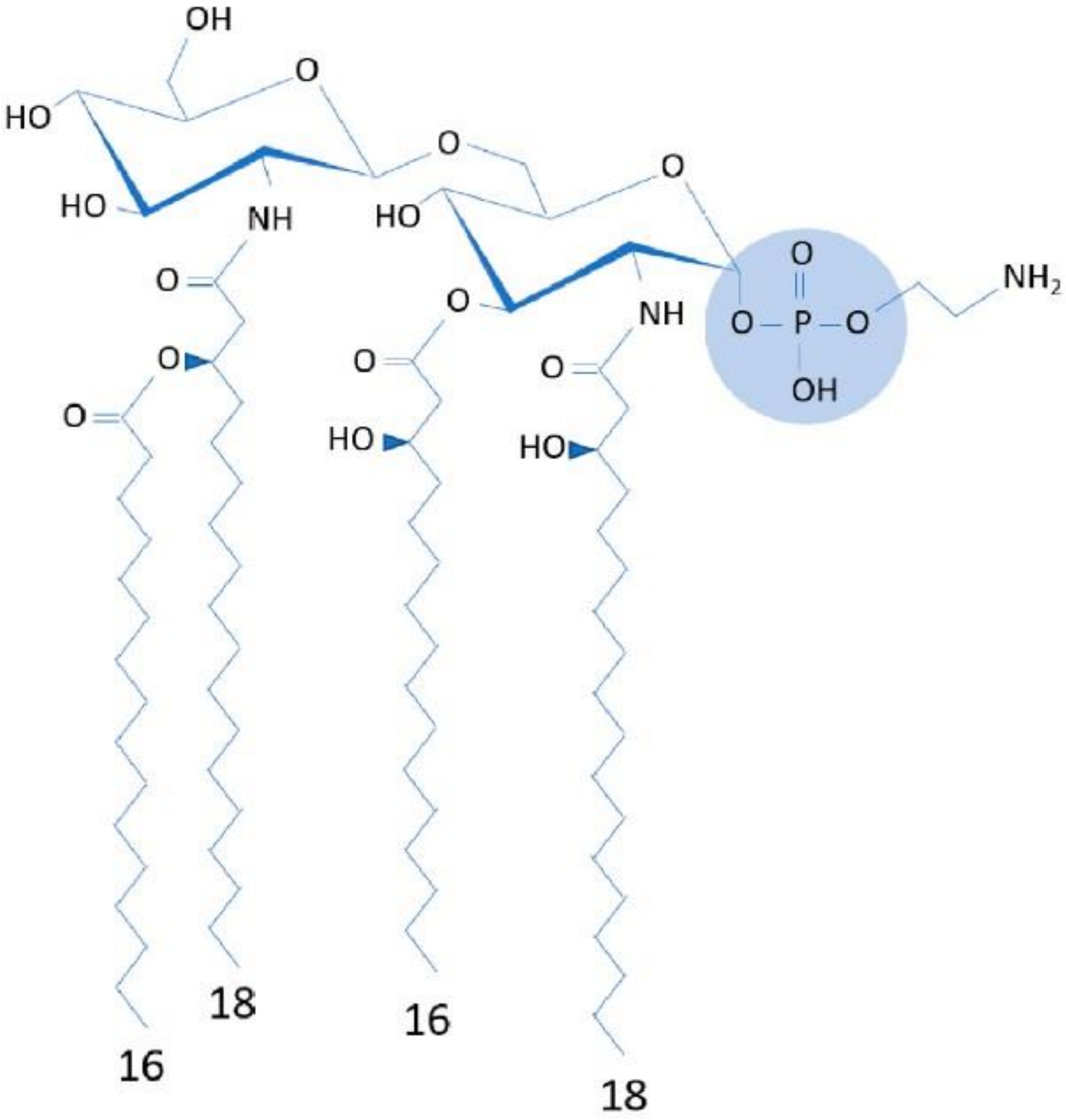

Figure S7. Chemical structures of LPS produced by *Helicobacter pylori* [47]

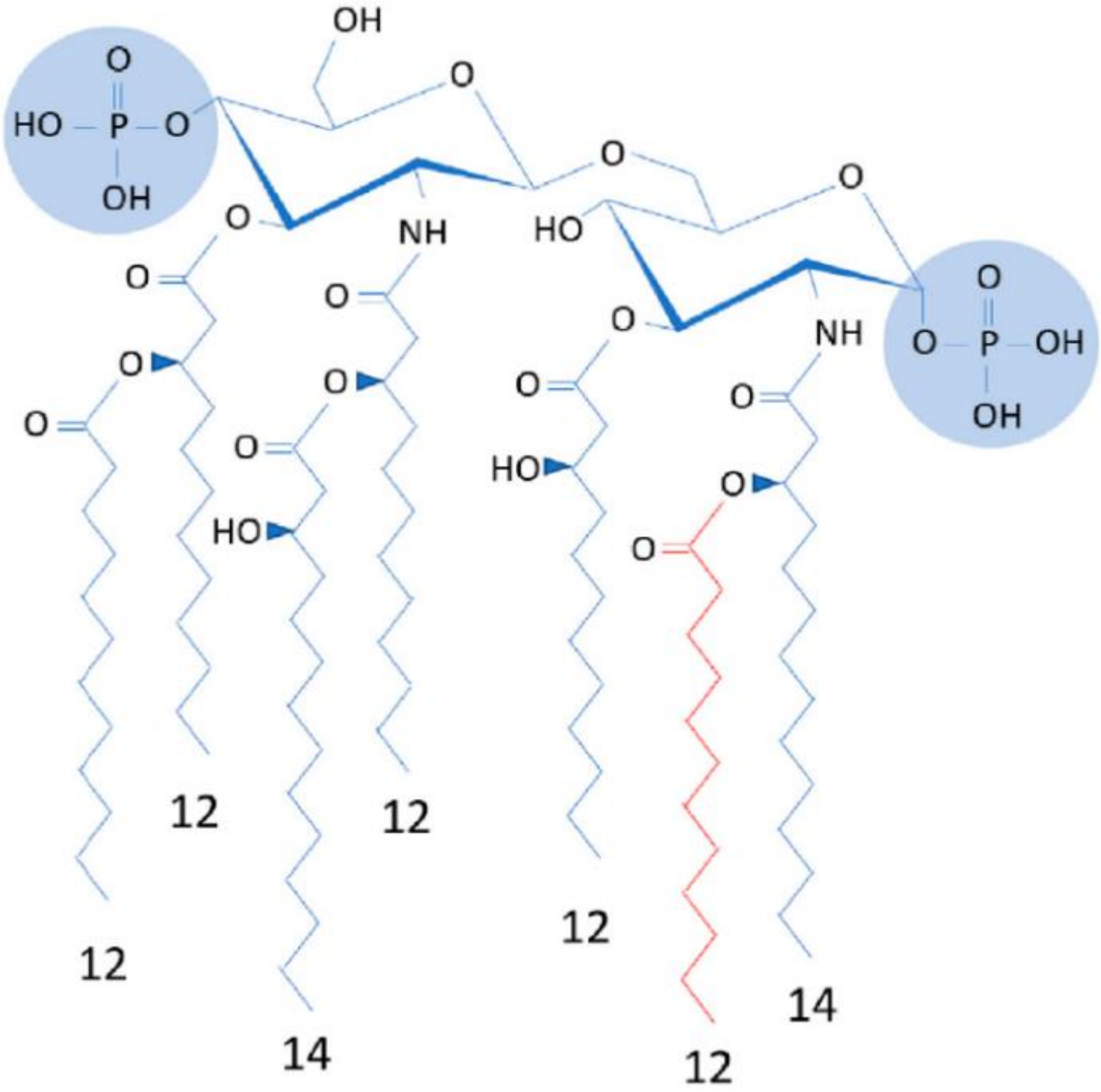

Figure S8. Chemical structures of LPS produced by *Acinetobacter baumannii* [47]

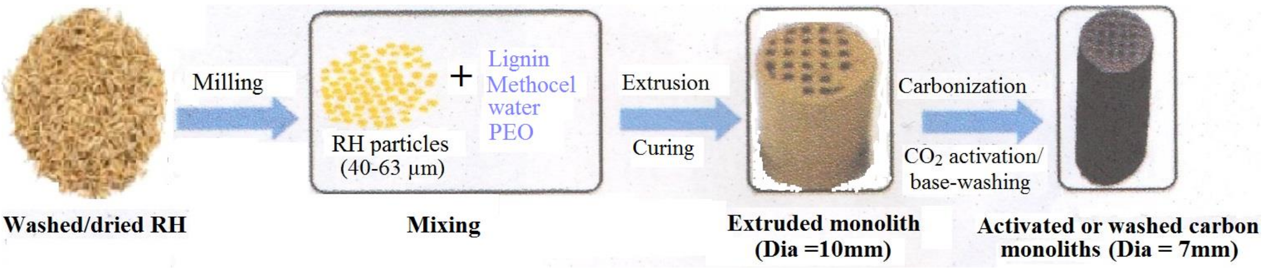

Figure S9. Scheme of the monoliths preparation techniques

References:

13 Durai, P.; Batool, M.; Choi, S. Structure and Effects of Cyanobacterial Lipopolysaccharides. *Mar. Drugs* **2015**, *13*, 4217–4230, doi:10.3390/md13074217.

43 Rezai et al. Bacterial cellulose to carbon, BioResources 15(2), 3408-3426. Bacterial cellulose as a carbon nano-fiber precursor: Enhancement of thermal stability and electrical conductivity. March **2020** *BioResources* 15(2):3408-3426Follow journal. DOI: 10.15376/biores.15.2.3408-3426

47 A.C. Fux, C. Melo, S. Michelini, B. Swartzwelter, A. Neusch, P. Italiani, M. Himly. Heterogeneity of Lipopolysaccharide as Source of Variability in Bioassays and LPS-Binding Proteins as Remedy *Int. J. Mol. Sci.* **2023**, 24, 8395. https://doi.org/10.3390/ijms24098395

51 M. Caroff, A. Novikov, Lipopolysaccharides: structure, function and bacterial identification. OCL **2020**, 27, 31. doi.org/10.1051/ocl/2020025
